# Matching biomedical ontologies based on formal concept analysis

**DOI:** 10.1186/s13326-018-0178-9

**Published:** 2018-03-19

**Authors:** Mengyi Zhao, Songmao Zhang, Weizhuo Li, Guowei Chen

**Affiliations:** 1 MADIS, Academy of Mathematics and Systems Science, Chinese Academy of Sciences, Beijing, People’s Republic of China; 2 University of Chinese Academy of Sciences, Beijing, People’s Republic of China

**Keywords:** Ontology matching, Formal concept analysis, Concept lattice

## Abstract

**Background:**

The goal of ontology matching is to identify correspondences between entities from different yet overlapping ontologies so as to facilitate semantic integration, reuse and interoperability. As a well developed mathematical model for analyzing individuals and structuring concepts, Formal Concept Analysis (FCA) has been applied to ontology matching (OM) tasks since the beginning of OM research, whereas ontological knowledge exploited in FCA-based methods is limited. This motivates the study in this paper, i.e., to empower FCA with as much as ontological knowledge as possible for identifying mappings across ontologies.

**Methods:**

We propose a method based on Formal Concept Analysis to identify and validate mappings across ontologies, including one-to-one mappings, complex mappings and correspondences between object properties. Our method, called FCA-Map, incrementally generates a total of five types of formal contexts and extracts mappings from the lattices derived. First, the token-based formal context describes how class names, labels and synonyms share lexical tokens, leading to lexical mappings (anchors) across ontologies. Second, the relation-based formal context describes how classes are in taxonomic, partonomic and disjoint relationships with the anchors, leading to positive and negative structural evidence for validating the lexical matching. Third, the positive relation-based context can be used to discover structural mappings. Afterwards, the property-based formal context describes how object properties are used in axioms to connect anchor classes across ontologies, leading to property mappings. Last, the restriction-based formal context describes co-occurrence of classes across ontologies in anonymous ancestors of anchors, from which extended structural mappings and complex mappings can be identified.

**Results:**

Evaluation on the Anatomy, the Large Biomedical Ontologies, and the Disease and Phenotype track of the 2016 Ontology Alignment Evaluation Initiative campaign demonstrates the effectiveness of FCA-Map and its competitiveness with the top-ranked systems. FCA-Map can achieve a better balance between precision and recall for large-scale domain ontologies through constructing multiple FCA structures, whereas it performs unsatisfactorily for smaller-sized ontologies with less lexical and semantic expressions.

**Conclusions:**

Compared with other FCA-based OM systems, the study in this paper is more comprehensive as an attempt to push the envelope of the Formal Concept Analysis formalism in ontology matching tasks. Five types of formal contexts are constructed incrementally, and their derived concept lattices are used to cluster the commonalities among classes at lexical and structural level, respectively. Experiments on large, real-world domain ontologies show promising results and reveal the power of FCA.

## Background

Ontologies aim to model domain conceptualizations so that applications built upon them can interoperate with each other by sharing the same meanings. Such knowledge sharing and reuse can be severely hindered by the fact that ontologies for the same domain are often developed for various purposes, differing in coverage, granularity, naming, structure and many other aspects. Ontology matching (OM) techniques aim to alleviate the heterogeneity by identifying correspondences across ontologies. Ontology matching can be performed at the element level and the structure level [1]. The former considers ontology classes and their instances independently, such as string-based and language-based techniques, whereas the latter exploits relations among entities, including graph-based and taxonomy-based techniques. Most ontology matching systems [2–8] adopt both element and structure level techniques to achieve better performance.

Life sciences is one of the most successful application areas of the Semantic Web technology, and many biomedical ontologies have been developed and utilized in real-world applications. These ontologies cover different yet overlapping domains and are often of large scale, including, for example, the Foundational Model of Anatomy (FMA) [9] and Adult Mouse Anatomy (MA) [10] for anatomy, National Cancer Institute Thesaurus (NCI) [11] for disease, and Systematized Nomenclature of Medicine-Clinical Terms (SNOMED-CT) [12] for clinical medicine. Moreover, efforts such as the Unified Medical Language System (UMLS) [13] integrate various biomedical systems so as to enhance their reuse and interoperability. For such biomedical domain ontologies, the annual Ontology Evaluation Alignment Initiative (OAEI) [14] sets three competition tracks, the Anatomy, the Large Biomedical Ontologies, and the Disease and Phenotype, which have attracted many state-of-the-art ontology matching systems [2–4, 7, 8] to challenge.

Among the first batch of OM algorithms and tools proposed in the early 2000s, FCA-Merge [15] distinguished in using the Formal Concept Analysis (FCA) formalism to derive mappings from classes sharing textual documents as their individuals. Proposed by Rudolf Wille [16], FCA is a well developed mathematical model for analyzing individuals and structuring concepts. FCA starts with a formal context consisting of a set of objects, a set of attributes, and their binary relations. Concept lattice, or Galois lattice, can be computed based on formal context, where each node represents a formal concept composed of a subset of objects (extent) with their common attributes (intent). The extent and the intent of a formal concept uniquely determine each other in the lattice. Moreover, the lattice represents a concept hierarchy where one formal concept becomes sub-concept of the other if its objects are contained in the latter.

Both ontologies and FCA aim at modeling “concepts” in hierarchical structures. The purpose of an ontology is to represent “a shared understanding of the domain of interest” [17] that can be queried and reasoned upon in an automated way. On the other hand, FCA is a conceptual clustering technique with solid mathematical foundations, allowing to derive concept hierarchies from datasets. Ontologies and FCA can complement each other, as analyzed in [18] from an application point of view. FCA can naturally be applied to constructing ontologies in ontology engineering [19–21], and is also widely used in data analysis, information retrieval, and knowledge discovery.

Following the steps of FCA-Merge, several OM systems continued to use FCA as well as its alternative formalisms, exploiting different entities as the sets of objects and attributes for constructing formal contexts [22–26]. FCA-OntMerge [23], for example, utilizes the classes of ontologies and their attributes to form its formal context, whereas in [22] the formal context is composed of ontology classes as objects and terms of a domain-specific thesaurus as attributes. Different types of formal contexts decide the information used for ontology matching, and we observed that some intrinsic and essential knowledge of ontology has not been involved yet, including both textual information within classes (e.g., class labels and synonyms) and relationships among classes (e.g., ISA, sibling, disjointedness relations, and properties and axioms).

This motivated the study in this paper, i.e., empowering FCA with as much as ontological information as possible for identifying and validating mappings across ontologies. Our method, called FCA-Map, incrementally generates a total of five types of formal contexts and extracts mappings from the lattices derived. First, the token-based formal context describes how class names, labels and synonyms share lexical tokens, leading to lexical mappings (anchors) across ontologies. Second, the relation-based formal context describes how classes are in taxonomic, partonomic and disjoint relationships with the anchors, leading to positive and negative structural evidence for validating the lexical matching. Third, after conflict repairing, the positive relation-based context can be used to discover structural mappings. Afterwards, the property-based formal context describes how object properties are used in axioms to connect anchor classes across ontologies, leading to property mappings. Last, the restriction-based formal context describes co-occurrence of classes across ontologies in anonymous ancestors of anchors, from which extended structural one-to-one mappings and complex mappings can be identified.

We participated in the three OAEI 2016 tracks related to the biomedical domain, and the results demonstrate the effectiveness of FCA-Map and its competitiveness with the OAEI top-ranked OM systems. FCA-Map is one of the three winners of the Disease and Phenotype track of the OAEI 2016 campaign. Our method is suitable for aligning large-scale domain ontologies with rich lexical and structural knowledge, due to a comprehensive construction of multiple FCA structures using names, hierarchies, properties, and axioms. This requires that ontologies provide meaningful lexical symbols and terms for classes, deep taxonomic hierarchies, and a large number of classes and expressive logical axioms specifying restrictions on properties linking classes. Such conditions can be satisfied by many ontologies in the biomedical domain, for which FCA-Map is effective and succeeds in discovering mappings that are missed by other OM systems.

The rest of the paper is organized as follows. We first introduce the basic definitions and characteristics of FCA. An overview of the FCA-Map method is presented, followed by five sections describing the five types of formal contexts and the derivation of mappings in detail. The evaluation section presents a comprehensive group of experiments, including the respective empirical results of the five steps as well as step-wise comparisons with counterparts. The evaluation also includes comparisons with OAEI 2016 top-ranked systems and previous FCA-based OM systems. Finally, we analyze in-depth the advantages and limitations of FCA-Map in contrast with other OM systems and FCA-based systems, and discuss the future work, followed by a conclusion.

## Preliminaries

Formal Concept Analysis (FCA) is a mathematical theory of data analysis based on applied lattice and order theory. FCA constructs formal contexts for objects and their attributes, and then derives concept hierarchical structures which constitute lattices. Formal context is defined as a triple $\mathbb {K}:=(G,M,I)$, where *G* is a set of objects, *M* a set of attributes, and *I* a binary relation between *G* and *M* in which *gIm* holds, i.e., (*g,m*)∈*I*, reads: object *g* has attribute *m* [27]. Formal contexts are often illustrated in binary tables, as exemplified by Table 1, where rows correspond to objects, columns to attributes, and a cell is marked with “ ×” if the object in its row has the attribute in its column. In Table 1, the marked cell represents that the animal listed in the row possesses the corresponding feature in the column.

**Table 1:** An example formal context $\mathbb {K}_{e}$

	Vertebrate	Mammal	Flying	Aquatic	Carnivorous
Elephant	×	×			
Dolphin	×	×		×	×
Porpoise	×	×		×	×
Hawk	×		×		×
Octopus				×	×

### **Definition 1**

*[*27*]* For subsets of objects and attributes *A*⊆*G* and *B*⊆*M*, derivation operators are defined as follows:

$$\begin{array}{*{20}l} A'&=\{ m\in M\ |\ gIm\ for \ all\ g\in A \} \\ B'&=\{g\in G\ |\ gIm\ for\ all\ m\in B\} \end{array} $$

*A*^′^ denotes the set of attributes common to the objects in *A*; *B*^′^ denotes the set of objects which have all the attributes in *B*.

A formal concept of context $ \mathbb {K} $ is a pair (*A,B*) consisting of extent *A*⊆*G* and intent *B*⊆*M* such that *A*=*B*^′^ and *B*=*A*^′^. $ \mathfrak {B}(\mathbb {K}) $ denotes the set of all formal concepts of context $ \mathbb {K} $. The partial order relation, namely subconcept-superconcept-relation, is defined as:

$$(A_{1},B_{1})\leq (A_{2},B_{2}):\Leftrightarrow A_{1}\subseteq A_{2}(\Leftrightarrow B_{1}\supseteq B_{2}) $$

Relation ≤ is called a hierarchical order of formal concepts. $ \mathfrak {B}(\mathbb {K}) $ ordered in this way is exactly a complete lattice, called the concept lattice and denoted by $ \mathfrak {\underline {B}}(\mathbb {K}) $.

**Fig. 1:**
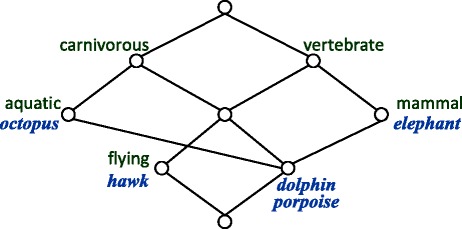
Concept lattice $ \mathfrak {\underline {B}}(\mathbb {K}_{e})$ with simplified labeling for the example formal context in Table 1

For an object *g*∈*G*, its *object concept* *γg*:=({*g*}^″^,{*g*}^′^) is the smallest concept in $ \mathfrak {\underline {B}}(\mathbb {K}) $ whose extent contains *g*. In other words, object *g* can generate formal concept *γg*. Symmetrically, for an attribute *m*∈*M*, its *attribute concept* *μm*:=({*m*}^′^,{*m*}^″^) is the greatest concept in $ \mathfrak {\underline {B}}(\mathbb {K}) $ whose intent contains *m*. In other words, attribute *m* can generate formal concept *μm*. For a formal concept (*A,B*), its *simplified extent* (*simplified intent*), denoted by *K*_*ex*_ (*K*_*in*_), is a minimal description of the concept. Each object (attribute) in *K*_*ex*_ (*K*_*in*_) can generate the formal concept (*A,B*). As a matter of fact, *K*_*ex*_ dose not occur in any descendant of (*A,B*) in $ \mathfrak {\underline {B}}(\mathbb {K}) $ and *K*_*in*_ dose not occur in any ancestor of (*A,B*) in $ \mathfrak {\underline {B}}(\mathbb {K}) $. Figure 1 shows the concept lattice of context $\mathbb {K}_{e}$ in Table 1. In the concept lattice diagrams in this paper, each node represents a formal concept labeled by its simplified intent and simplified extent, the latter being given in italics. A line connecting two nodes represents that the lower formal concept is a subconcept of the upper concept. The node at the top represents suprema whose extent is the set of all objects, whereas the node at the bottom is infima whose intent is the set of all attributes.

## Methods

Given two ontologies, FCA-Map builds formal contexts and uses the derived concept lattices to cluster the commonalities among ontology entities including classes and object properties, at lexical level and structural level, respectively. Concretely, FCA-Map performs step-by-step as follows, where a total of five types of contexts are constructed.

Step 1 **Acquiring anchors lexically.** Based on class names, labels and synonyms, the token-based formal context is constructed, and from its derived concept lattice, a group of lexical mappings between classes across ontologies can be extracted, called lexical anchors $ \mathcal {A}_{class}^{0} $.
Step 2 **Validating anchors structurally.** Based on $ \mathcal {A}_{class}^{0}$, *ISA* and *PART-OF* hierarchies and disjointness axioms, the relation-based formal context is constructed, and from its derived concept lattice, positive and negative structural evidence of anchors can be extracted. Moreover, an enhanced alignment $ \mathcal {A}_{class}^{1} $ without any conflicts among anchors is obtained.
Step 3 **Discovering structural matches.** Based on $ \mathcal {A}_{class}^{1} $ and *ISA* and *PART-OF* hierarchies, the positive relation-based formal context is constructed, and from its derived concept lattice, structural matches among classes across ontologies can be identified, augmenting $ \mathcal {A}_{class}^{1}$ to alignment $ \mathcal {A}_{class}^{2}$.
Step 4 **Acquiring property mappings.** Based on $ \mathcal {A}_{class}^{2} $ and axioms specifying that object properties hold between instances of class mappings, the property-based formal context is constructed, and from its derived concept lattice, a group of mappings among properties across ontologies $ \mathcal {A}_{property} $ can be extracted.
Step 5 **Identifying extended and complex mappings.** Based on $ \mathcal {A}_{property} $, $ \mathcal {A}_{class}^{2} $ and axioms specifying restrictions on how to use properties with respect to classes, the restriction-based formal context is constructed, and from its derived concept lattice, extended structural mappings among classes across ontologies $ \mathcal {A}_{class}^{3}$ can be extracted, including one-to-one mappings and complex mappings where a class is identified to correspond to a semantic expression composed of classes and properties in another ontology.

To illustrate every step of FCA-Map, we use parts of four matching tasks from the Anatomy track and the Large Biomedical Ontologies track of OAEI 2016, shown in Table 2, as running examples in the subsequent sections. MA, NCI, FMA, and SNOMED-CT are all real-world, biomedical ontologies and the versions used are the OWL files provided by OAEI. These matching tasks use small fragments of the corresponding ontologies, whose proportions are listed in Table 2.

**Table 2:** Matching tasks of fragment ontologies of the OAEI 2016 Anatomy track and the Large Biomedical Ontologies track

Matching task	Number of classes in *O*_1_	Number of classes in *O*_2_
MA-NCI	2744 (100% of MA)	3304 (5% of NCI)
FMA-NCI	3696 (5% of FMA)	6488 (10% of NCI)
FMA-SNOMED	10157 (13% of FMA)	13412 (5% of SNOMED)
SNOMED-NCI	51128 (17% of SNOMED)	23958 (36% of NCI)

## Constructing the token-based formal context to acquire lexical anchors

Most OM systems rely on lexical matching as initiation due to the fact that classes sharing names across ontologies quite likely represent the same entity in the domain of interest. FCA-Map, rather than using lexical and linguistic analyzing techniques, generates a formal context at the lexical level and obtains mappings from the lattice derived from the context. Concretely, names of ontology classes as well as their labels and synonyms, when available, are exploited after normalization that includes inflection, tokenization, stop word elimination^1^, and punctuation elimination. The token-based formal context for ontology matching is defined as follows.

### **Definition 2**

The token-based formal context for ontology matching is a triple $\mathbb {K}_{lex}:=(G_{lex},M_{lex},I_{lex})$, where objects *G*_*lex*_ is the set of strings each corresponding to a name, label, or synonym of classes in two source ontologies, attributes *M*_*lex*_ is the set of tokens in these strings, and binary relation (*g,m*)∈*I*_*lex*_ holds when string *g* contains token *m*, or a synonym or lexical variation of *m*.

**Fig. 2:**
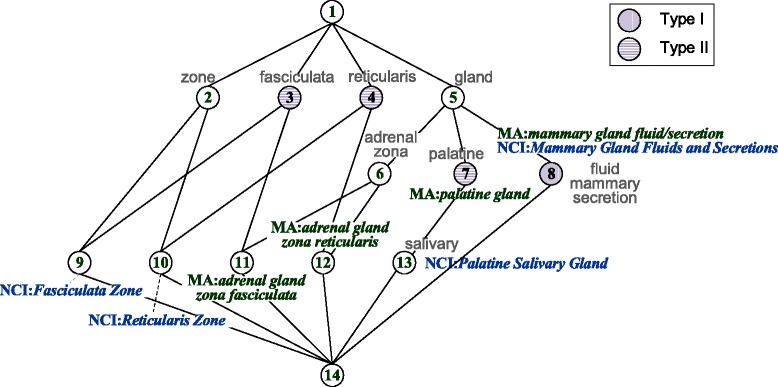
Concept lattice with simplified labeling derived from $ \mathbb {K}_{lex} $ in Table 3

We use the UMLS Sub-Term Mapping Tools [28] to access synonyms and the UMLS SPECIALIST Lexicon [29] to access lexical variations of biomedical terms. Table 3 shows $ \mathbb {K}_{lex} $ of a small part of MA and NCI, and its derived concept lattice is displayed in Fig. 2. For each formal concept derived, in addition to the strings in its extent, we are also interested in the classes that these strings come from, and we call them *class-origin extent*. For example, in Fig. 2, the *class-origin extent* of formal concept by node 8 is {MA:*mammary gland fluid/secretion*, NCI:*Breast Fluid or Secretion* } since in NCI, “*Mammary Gland Fluids and Secretions*” is a synonym of class NCI:*Breast Fluid or Secretion*.

**Table 3:** Token-based formal context $ \mathbb {K}_{lex} $ of a small part of MA and NCI

	Gland	Adrenal	Zona	Zone	Fasciculata	Reticularis	Salivary	Palatine	Mammary	Secretion	Fluid
MA:*palatine gland*	×							×			
MA:*adrenal gland zona fasciculata*	×	×	×		×						
MA:*adrenal gland zona reticularis*	×	×	×			×					
MA:*mammary gland fluid/secretion*	×								×	×	×
NCI:*Palatine Salivary Gland*	×						×	×			
NCI:*Fasciculata Zone*				×	×						
NCI:*Reticularis Zone*				×		×					
NCI:*Mammary Gland Fluids and Secretions*	×								×	×	×

An essential feature of FCA is the duality between a set of objects and their attributes. The more attributes demanded, the fewer objects can meet the requirements. In the case of the token-based formal concept, the more common tokens occurring in its intent, the fewer strings the extent contains, and the more possibly for the classes in *class-origin extent* to be matched. This is to say that cardinality of the extent can reflect how similar the strings are, thus classes from different source ontologies in a smaller-sized *class-origin extent* can be considered as a mapping with higher confidence. Practically, we restrict our attention to formal concepts whose *simplified extent* or *class-origin extent* contains exactly two strings or classes across ontologies, and extract two types of lexical anchors, namely ***Type I anchor*** for the exact match, and ***Type II anchor*** for the partial match, respectively. On the other hand, note that cardinality of the intent cannot be used to measure the similarity of strings. For example, MA:*nerve* and NCI:*Nerve*, which is a match, share only one token, whereas MA:*left lung respiratory bronchiole* and NCI:*Right Lung Respiratory Bronchiole*, not a match, share three tokens.

***Type I anchor***. *Simplified extent* *K*_*ex*_ of the formal concept contains exactly two strings from classes across ontologies. This indicates that the two strings are composed of the same or synonymous tokens, thus the corresponding classes are extracted to be a match, as exemplified by 〈MA:*mammary gland fluid/secretion*, NCI:*Breast Fluid or Secretion* 〉 through formal concept of node 8 in Fig. 2 whose *K*_*ex*_ has two strings, one from MA and the other NCI.

***Type II anchor.*** The *class-origin extent* of the formal concept contains exactly two classes across ontologies and *simplified extent* *K*_*ex*_ contains strings from at most one source ontology. Here the strings share tokens in the intent rather than composed of the same or synonymous tokens. For example, 〈MA:*adrenal gland zona fasciculata*, NCI:*Fasciculata Zone* 〉 is extracted from node 3 in Fig. 2, due to the common token “fasciculata” which exists solely in these two classes. And 〈MA:*palatine gland*, NCI:*Palatine Salivary Gland* 〉 is identified as an anchor from node 7, due to the common tokens “palatine” and “gland” which co-exist solely in these two classes.

## Constructing the relation-based formal context to validate lexical anchors

Structural relationships of ontologies are exploited to validate the matches obtained at the lexical level. One of our previous studies [30] proposed using positive and negative structural evidence among anchors for the purpose of validation. More precisely, classes of one anchor sharing relationships to classes in another anchor can be seen as their respective positive evidence. On the other hand, negative structural evidence refers to the conflict based on the disjointedness relationships between classes. In FCA-Map, we build the relation-based formal context, defined as follows, to obtain both positive and negative structural evidence for lexical anchors. Specifically, we exploit the taxonomic, partonomic and disjoint relationships which are common in biomedical ontologies. Both explicitly represented and inferred semantic relations are used in our method.

### **Definition 3**

The relation-based formal context for ontology matching is a triple $\mathbb {K}_{rel}:=(G_{rel},M_{rel},I_{rel})$, where objects *G*_*rel*_ is the set of all classes in two source ontologies, and attributes *M*_*lex*_ is the lexical anchors prefixed with four kinds of relationships, i.e., *ISA*, *SIBLING-WITH*, *PART-OF*, and *DISJOINT-WITH*, labeled by “(ISA)”, “(SIB)”, “(PAT)”, and “(I-D)” (or “(D-I)”), respectively. Binary relation (*g,m*)∈*I*_*rel*_ holds if *g* in its ontology has the relationship *ISA*, *SIBLING-WITH*, *PART-OF*, or *DISJOINT-WITH* (as in the prefix of *m*) with the class in anchor *m*.

The relation-based formal context $ \mathbb {K}_{rel} $ of a small part of MA and NCI is displayed in Table 4. For instance, MA:*periodontal ligament* and NCI:*Periodontium* are subclasses of MA:*ligament* and NCI:*Ligament*, respectively, thus (MA:*periodontal ligament*, (ISA) 〈MA:*ligament*, NCI:*Ligament* 〉)∈*I*_*rel*_ and (NCI:*Periodontium*, (ISA) 〈MA: *ligament*, NCI:*Ligament* 〉) ∈*I*_*rel*_ hold. Moreover, MA:*adipose tissue* is a subclass of MA:*organ system* whereas NCI:*Adipose Tissue* is disjoint with NCI:*Organ System*, thus (MA:*adipose tissue*, (I-D) 〈MA:*organ system*, NCI:*Organ system* 〉)∈*I*_*rel*_ and (NCI:*Adipose Tissue*, (I-D) 〈MA:*organ system*, NCI:*Organ system* 〉)∈*I*_*rel*_ hold.

**Table 4:** Relation-based formal context $ \mathbb {K}_{rel} $ of a small part of MA and NCI

	(ISA) 〈MA:*ligament*, NCI:*Ligament* 〉	(I-D) 〈MA:*organ system*, NCI:*Organ System* 〉	(SIB) 〈MA:*adipose tissue*, NCI:*Adipose Tissue* 〉	(SIB) 〈MA:*larynx ligament*, NCI:*Laryngeal Ligament* 〉	(PAT) 〈MA:*larynx*,NCI:*Larynx* 〉
MA:*ligament*		×	×		
MA:*periodontal ligament*	×	×		×	
MA:*auricular ligament*	×	×		×	
MA:*adipose tissue*		×			
MA:*larynx ligament*	×	×			×
NCI:*Ligament*		×			
NCI:*Periodontium*	×	×		×	
NCI:*Broad Ligament*	×	×		×	
NCI:*Adipose Tissue*		×			
NCI:*Laryngeal Ligament*	×	×			×

**Fig. 3:**
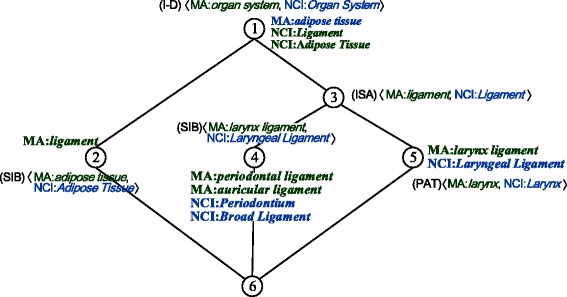
Concept lattice of $ \mathbb {K}_{rel} $ with simplified labeling

The derived concept lattice $ \mathbb {K}_{rel} $ of a small part of MA and NCI is illustrated in Fig. 3. Formal concepts whose extents include both classes in an anchor indicate structural evidence, defined as follows.

### **Definition 4**

In the derived concept lattice of the relation-based formal context $\mathbb {K}_{rel}$, if a formal concept (*A,B*) satisfies that its extent *A* includes both classes in the same anchor **a**, then for anchors in its intent *B* with label “(ISA)”, “(SIB)” or “(PAT)”, **a** is a positive evidence; and for anchors in its intent *B* with label “(I-D)” or “(D-I)”, **a** is a negative evidence.

For example, in the extent of node 3 in Fig. 3, 〈MA:*periodontal ligament*, NCI:*Periodontium* 〉 and 〈MA:*larynx ligament*, NCI:*Laryngeal Ligament* 〉, two anchors acquired lexically, are positive evidences to anchor 〈MA:*ligament*, NCI:*Ligament* 〉 with label “(ISA)” in the intent, and negative evidences to anchor 〈MA:*organ system*, NCI:*Organ System* 〉 with label “(I-D)”. We use *P*(**a**) and *N*(**a**) to denote the sets of positive and negative structural evidence of anchor **a**, respectively, whose cardinalities are called the *support degree* and *conflict degree* of anchor **a**. FCA-Map utilizes all the positive evidence sets $\mathcal {P}$ and negative evidence sets $\mathcal {N}$ to eliminate incorrect lexical anchors and retain the correct ones, as follows.

*Conflict repairing.* The negative evidence leads to conflicts among anchors, for which FCA-Map repairs in a greedy way, i.e., eliminating the conflict-causing anchors iteratively until $\mathcal {N}$ becomes empty. At each iteration, anchor **a** having the least negative evidence set, i.e., the smallest *conflict degree*, is selected. For every anchor **b** in *N*(**a**), if *conflict degree* of **b** is greater than **a**, eliminate **b**; otherwise, compare the *support degree* of **a** and **b**, and eliminate the one with smaller *support degree*.

*Anchor screening.* Anchors having no positive structural evidence according to the updated $\mathcal {P}$ are either caused by the structural isolation of classes, or simply mismatches. FCA-Map screens anchors based on both lexical and structural evidence, and ***Type II anchors*** without positive evidence are eliminated.

## Constructing the positive relation-based formal context to discover structural matches

After conflict repairing and screening, anchors retained are those supported both lexically and structurally. Based on the enhanced alignment, FCA-Map goes further to build the positive relation-based formal context aiming to identify new, structural mappings. The way positive relation-based formal context $\mathbb {K}_{posRel} $ constructed is similar to $\mathbb {K}_{rel}$, i,e., using classes in two source ontologies as object set and anchors prefixed with relationship labels as attribute set. Concretely, five kinds of relationships are considered, *ISA*, *SUPERCLASS-OF*, *SIBLING-WITH*, *PART-OF*, and *HAS-PART*, where disjointedness relationship is no longer necessary. For the derived formal concepts, we restrict our attention to those with classes across ontologies in the *simplified extent*, and both *one-to-one* mappings and *complex* mappings can be identified.

***One-to-one*** **structural mappings** are extracted from the formal concepts whose *simplified extent* exactly contains two classes across ontologies. Although most of the mappings extracted this way have already been identified at the lexical level, new additional matches emerge, as exemplified by 〈MA:*hindlimb bone*, NCI:*Bone of the Lower Extremity* 〉.

***Complex*** **mappings** are traced from the formal concepts whose *simplified extents* contain more than two classes from different source ontologies. It means that these classes share the same structural relationships to anchors in the intent. Such classes may compose a complex mapping, as elaborated in the following.

1. *One-to-group mappings*. The *simplified extent* contains only one class from one source ontology and multiple classes from the other source ontology. For example, MA:*inferior suprarenal vein* can be mapped to the group of concepts {NCI:*Left Suprarenal Vein*, NCI:*Right Suprarenal Vein* } as the three concepts are contained within one *simplified extent* that has no more classes. This one-to-group mapping comes from the difference in granularity between MA and NCI.
2. *Group-to-group mappings*. The *simplified extent* contains multiple classes from different source ontologies, respectively. For example, two groups of concepts {MA: *sacral vertebra 1*, MA:*sacral vertebra 2*, MA:*sacral vertebra 3*, MA:*sacral vertebra 4* } and {NCI:*S1 Vertebra*, NCI:*S2 Vertebra*, NCI:*S3 Vertebra*, NCI:*S4 Vertebra*, NCI:*S5 Vertebra* } can be mapped as these classes are contained in one *simplified extent* that has no more classes. This group-to-group mapping represents the difference between mouse and human anatomy.

In all the four matching tasks of Table 2, such complex mappings can be identified, as shown in Table 5, where the classes within one mapping are of the same type, thus the logical constructor used in the semantic expressions is disjunction. Note that no extra operations are needed in FCA-Map for identifying such complex mappings as they and the one-to-one mappings are implied similarly in the formal concepts derived from the positive relation-based formal context.

**Table 5:** Some one-to-group and group-to-group mappings discovered by the positive relation-based formal contexts

	Classes	Semantic expressions
MA	Inferior suprarenal vein	Inferior suprarenal vein
NCI	Left Suprarenal Vein, Right Suprarenal Vein	(Left Suprarenal Vein ⊔ Right Suprarenal Vein)
FMA	T helper cell type 1, T helper cell type 2	(T helper cell type 1 ⊔ T helper cell type 2)
SNOMED	T helper subset 1 cell, T helper subset 2 cell	(T helper subset 1 cell ⊔ T helper subset 2 cell)
FMA	First sacral spinal ganglion,	(First sacral spinal ganglion
	Second sacral spinal ganglion,	⊔ Second sacral spinal ganglion
	Third sacral spinal ganglion	⊔ Third sacral spinal ganglion)
SNOMED	S1 spinal ganglion,	(S1 spinal ganglion
	S2 spinal ganglion,	⊔ S2 spinal ganglion
	S3 spinal ganglion	⊔ S3 spinal ganglion)
SNOMED	Simian foamy virus,	(Simian foamy virus
	Chimpanzee foamy virus,	⊔ Chimpanzee foamy virus
	Chimpanzee foamy virus human isolate	⊔ Chimpanzee foamy virus human isolate)
NCI	Foamy Retrovirus	Foamy Retrovirus
SNOMED	Malignant teratoma of undescended testis	Malignant teratoma of undescended testis
NCI	Stage I Immature Testicular Te ratoma,	(Stage I Immature Testicular Te ratoma
	Stage II Immature Testicular Teratoma	⊔ Stage II Immature Testicular Teratoma
	Stage III Immature Testicular Teratoma,	⊔ Stage III Immature Testicular Teratoma)

## Constructing the property-based formal context to acquire property mappings

Properties across ontologies tend to differ greatly in names, even for ontologies of the same domain [30]. Thus, we utilize the structural rather than lexical information to obtain property mappings. Axioms specifying what properties are used to link the individuals of anchors in respective ontologies are the core for identifying the commonalities among properties.

### **Definition 5**

The property-based formal context for ontology matching is a triple $\mathbb {K}_{pro}:=(G_{pro},M_{pro},I_{pro})$, where objects *G*_*pro*_ is the set of all object properties in two source ontologies, and attributes *M*_*pro*_ is the pairs of one-to-one class mappings. Binary relation (*g,m*)∈*I*_*pro*_ holds where *m*=<(*C*_*Ai*_,*C*_*Bi*_),(*C*_*Aj*_,*C*_*Bj*_)>,*i*≠*j*, if axiom $C_{Ai} \sqsubseteq \exists g.C_{Aj} $ or $C_{Ai} \sqsubseteq \forall g.C_{Aj} $ ($C_{Bi} \sqsubseteq \exists g.C_{Bj} $ or $C_{Bi} \sqsubseteq \forall g.C_{Bj} $) is asserted or can be inferred within one source ontology.

The property-based formal context $ \mathbb {K}_{pro} $ of a small part of SNOMED and NCI is displayed in Table 6. Take the second column of Table 6 for example. The two cells are marked because axioms *Benign neoplasm of buccal mucosa*$ \sqsubseteq \exists $*Finding site*.*Buccal mucosa* and *Benign Buccal Mucosa Neoplasm*$ \sqsubseteq \forall $Disease Has Primary Anatomic Site.*Buccal Mucosa* can be inferred in SNOMED and NCI, respectively.

**Table 6:** Property-based formal context $ \mathbb {K}_{pro} $ of a small part of SNOMED and NCI

	<〈SNOMED:*Benign neoplasm of buccal mucosa*, NCI:*Benign Buccal Mucosa Neoplasm* 〉, 〈SNOMED:*Buccal mucosa*, NCI:*Buccal Mucosa* 〉>	<〈SNOMED:*Synovioma benign*, NCI:*Benign Synovial Neoplasm* 〉, 〈SNOMED:*Soft tissues*, NCI:*Soft Tissue* 〉>	<〈SNOMED:*Phocomelia of upper limb NOS*, NCI:*Phocomelia of the Upper Limb* 〉, 〈SNOMED:*Upper extremity part*, NCI:*Upper Extremity Part* 〉>	<〈SNOMED:*Bowenoid papulosis*, NCI:*Bowenoid Papulosis* 〉, 〈SNOMED:*Human papilloma virus infection*, NCI:*Human Papilloma Virus Infection* 〉>	<〈SNOMED:*Insulin coma*, NCI:*Insulin Coma* 〉, 〈SNOMED:*Hypoglycemia*, NCI:*Hypoglycemia* 〉>	<〈SNOMED:*Laparoscopy*, NCI:*Laparoscopy* 〉, 〈SNOMED:*Endoscope device*, NCI:*Endoscope* 〉>
SNOMED:*Finding site*	×	×	×			
SNOMED:*Due to*				×	×	
SNOMED:*Using device*						×
NCI:*Disease Has Primary Anatomic Site*	×	×				
NCI:*Disease May Have Associated Disease*				×		

**Fig. 4:**
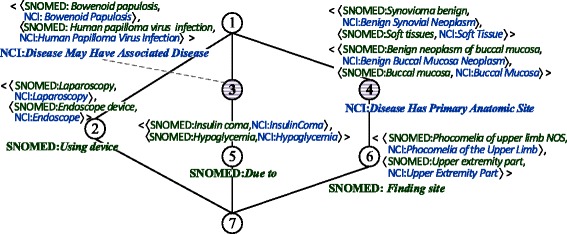
Concept lattice of $ \mathbb {K}_{pro} $ with simplified labeling

The derived concept lattice of $ \mathbb {K}_{pro} $ of a small part of SNOMED and NCI is illustrated in Fig. 4. We can extract property mappings from the formal concepts whose extents contain exactly two properties across ontologies. This means that they are used to connect the same pairs of mappings. For example, 〈SNOMED:Finding site, NCI:Disease Has Primary Anatomic Site 〉 is extracted from node 4 in Fig. 4.

## Constructing the restriction-based formal context to acquire extended and complex mappings

**Fig. 5:**
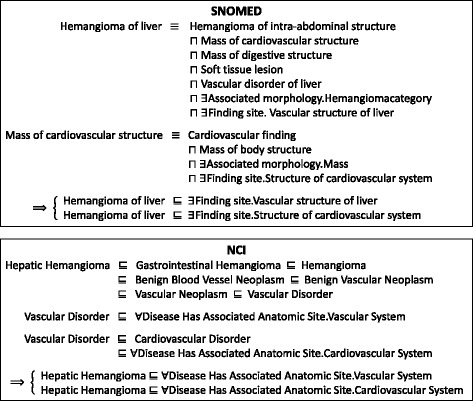
Inferring anonymous ancestors of SNOMED:*Hemangioma of liver* and NCI:*Hepatic Hemangioma*

With the availability of property mappings, we can start exploiting anonymous classes in ontologies, i.e., restrictions on how to use properties with respect to classes. An axiom with a named class at the left-hand side and a restriction at the right-hand actually defines a necessary condition for the class, and the condition becomes necessary and sufficient in equivalent axioms. When two classes in an anchor have necessary conditions (restrictions) described by the same property, the two classes specified in the restrictions, i.e., fillers of the property, could possibly be a match across ontologies. We illustrate this by a validated anchor 〈SNOMED:*Hemangioma of liver*, NCI:*Hepatic Hemangioma* 〉. All the anonymous ancestors of these two classes in SNOMED and NCI, respectively, are listed in Table 7. They are either asserted or inferred, as shown in Fig. 5. Since 〈SNOMED:Finding site, NCI:Disease Has Associated Anatomic Site 〉 is a property mapping, one can see that the fillers of the properties imply some correspondences across two ontologies. We pair fillers in anonymous ancestors of the two classes in anchor, denoted as $ \mathcal {FP}$. In the case of anchor 〈SNOMED:*Hemangioma of liver*, NCI:*Hepatic Hemangioma* 〉, 16 such pairs can be generated. We utilize these potential matches to construct a FCA formal context so as to confirm the correct mappings.

**Table 7:** Anonymous ancestors of SNOMED:*Hemangioma of liver* and NCI:*Hepatic Hemangioma*

Classes in an anchor	Anonymous ancestors
SNOMED:*Hemangioma of liver*	∃ Finding site.Structure of cardiovascular system
	∃ Finding site.Blood vessel structure
	∃ Finding site.Vascular structure of liver
	∃ Finding site.Liver structure
NCI:*Hepatic Hemangioma*	∀ Disease has associated anatomic site.Cardiovascular system
	∀ Disease has associated anatomic site.Vascular system
	∀ Disease has associated anatomic Site.Blood vessel
	∀ Disease has associated anatomic Site.Liver

### **Definition 6**

The restriction-based formal context for ontology matching is a triple $\mathbb {K}_{res}:=(G_{res},M_{res},I_{res})$, where objects *G*_*res*_ is the set of all classes in one source ontology, and attributes *M*_*res*_ is the set of all classes in the other source ontology. Binary relation (*g,m*)∈*I*_*res*_ holds if $ (g,m)\in \mathcal {FP}$, where $\mathcal {FP}$ denotes the set of pairs (*D,E*) from axiom $C_{A} \sqsubseteq \exists g.D $ (or $C_{A} \sqsubseteq \forall g.D $) in one ontology and axiom $C_{B} \sqsubseteq \exists h.E $ (or $C_{A} \sqsubseteq \forall h.E $) in the other ontology where 〈*C*_*A*_,*C*_*B*_〉 is a class mapping and 〈*g,h*〉 a property mapping.

**Fig. 6:**
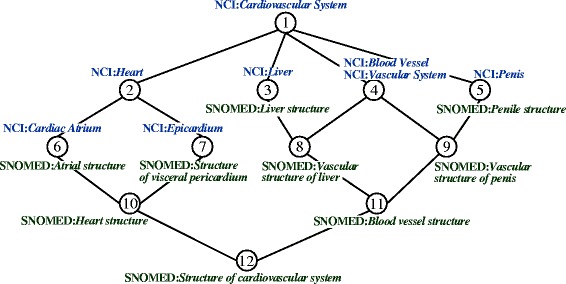
Concept lattice of $ \mathbb {K}_{res}$ with simplified labeling

Table 8 shows $ \mathbb {K}_{res} $ of a small part of SNOMED and NCI, where the gray area corresponds to Table 7. The derived concept lattice of $ \mathbb {K}_{res} $ of a small part of SNOMED and NCI is illustrated in Fig. 6. Mappings can be extracted from the formal concepts according to the *simplified extent* *K*_*ex*_ and *simplified intent* *K*_*in*_.

**Table 8:** Restriction-based formal context $ \mathbb {K}_{res} $ of a small part of SNOMED and NCI

	

For a formal concept (*A,B*) with non-empty simplified intent and simplified extent, *K*_*in*_ represents the attributes uniquely introduced by (*A,B*) compared with all its ancestors in the lattice. Similarly, *K*_*ex*_ is the set of objects uniquely introduced by (*A,B*) compared with all its descendants. Hence, *K*_*in*_ and *K*_*ex*_ are introduced by formal concept (*A,B*) at the same time, in other words, the objects in *K*_*ex*_ specifically embody the attributes in *K*_*in*_; and the attributes in *K*_*in*_ describe the most particular characteristics of the objects in *K*_*ex*_. In the case of the restriction-based concept lattice, if both *K*_*ex*_ and *K*_*in*_ of a formal concept contain exactly one class, then it means that these two classes always occur at the same time as fillers of the same properties in anonymous ancestors of anchors. They are more likely a match than other filler pairs in $\mathcal {FP}$ that are also present across the intent and extent of the same formal concept. For example, node 7 in Fig. 6 represents a formal concept with intent {NCI:*Cardiovascular System*, NCI:*Heart*, NCI:*Epicardium* } and extent {SNOMED:*Structure of visceral pericardium*, SNOMED:*Heart structure*, SNOMED:*Structure of cardiovascular system* }. Its simplified intent is {NCI:*Epicardium* } and its simplified extent {SNOMED:*Structure of visceral pericardium* }, indicating that these two classes are always used as fillers at the same time, i.e., in the restrictions about the same properties for the same anchor classes across ontologies. For other pairs of classes across the intent and extent of node 7 in Fig. 6, their two classes may occur as fillers at the same time but not always. Thus 〈SNOMED:*Structure of visceral pericardiumis*,NCI:*Epicardium* 〉 is extracted to be a match. Similarly, node 6 in Fig. 6 yields match 〈SNOMED:*Atrial structure*,NCI:*Cardiac Atrium* 〉.

**Fig. 7:**
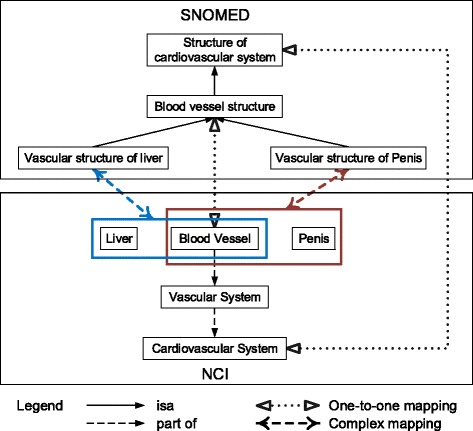
Complex mappings discovered from the lattice in Fig. 6

There are formal concepts in the restriction-based lattice that have an empty simplified intent (extent) and a non-empty simplified extent (intent), indicating the difference in class hierarchies and expressions of axioms across two ontologies. Rather than one-to-one mappings, complex mappings might be implied in such cases. For example, node 8 in Fig. 6 has an empty *K*_*in*_ whereas its *K*_*ex*_ is {SNOMED:*Vascular structure of liver* }. Instead of one class, there may be a complex combination of NCI classes in the complete intent of node 8 that corresponds to {SNOMED:*Vascular structure of liver* }. Under a manual review, a complex mapping is determined, as illustrated in Fig. 7.

## Results

To evaluate the effectiveness of FCA-Map, we conduct experiments on three OAEI 2016 biomedical tracks, the Anatomy, the Large Biomedical Ontologies, and the Disease and Phenotype. Additionally, we run FCA-Map on the Conference track to test its performance on a relatively general-purpose domain. The versions used are the OWL files of the ontologies provided by OAEI 2016, and the precision, recall and F-measure values listed in the subsequent subsections are computed based on the reference alignments provided by OAEI. In the Large Biomedical Ontologies track, the references are extracted from the UMLS Metathesaurus mappings, which, despite of being created by domain experts under comprehensive auditing protocols, lead to unsatisfiability when integrated with source ontologies [31]. Those incoherence-causing mappings are identified by OAEI and denoted as the “Unknown” category, i.e., neither correct nor incorrect when evaluating the alignment, thus ignored.

The evaluation consists of a total of ten experiments. In the following, we first present the results of Step 1 of FCA-Map, the token-bases lexical matching, followed by an empirical comparison with another token-based lexical method TFIDF. We then present the results of Step 2 of FCA-Map, structural validation, followed by an empirical comparison with the work on incoherence detection and repairing of ontology mappings. Third, we present the results of Step 3 of FCA-Map, followed by an empirical comparison with another structural matching method. Afterwards, the results of Step 4 of property matching and then Step 5 of extended structural matching in FCA-Map are presented. These experiments are conducted on matching tasks in Table 2. Furthermore, FCA-Map is compared with the OAEI 2016 top-ranked systems on all matching tasks in the three biomedical tracks and the Conference track, where the runtimes are also analyzed. Last, we compare with the innovative FCA-Merge, the first OM system that proposes to use the FCA formalism.

Even for the ontologies in Table 2 that only take a small portion of their original, complete systems, the formal contexts constructed are of large size, resulting in complex structures of the concept lattices derived. In order to avoid generating redundant information, Galois Sub-hierarchy (GSH) [32], a polynomial-sized representation of concept lattice that preserves the most pertinent information, is utilized in FCA-Map. Concretely, we use FCAlib [33] to derive concept lattices (GSH) from formal contexts. FCAlib is an open-source, extensible library for FCA tool developers, and FCA-Map is implemented in Java. All the experiments were performed on a desktop computer with Intel^Ⓡ^ Core^TM^ i7-2600 (3.4GHz) and 32GB RAM in Java 1.8.

### The results of the token-based lexical matching

FCA-Map starts with building the token-based formal context so as to identify the lexical correspondence among classes in two source ontologies. The results of such lexical anchors are summarized in Table 9. One can see that most of the lexical anchors are of ***Type I***, i.e., the name, synonym or label of one class is the same as another class. For example, MA:*cortical layer II* and NCI:*External Granular Layer* are extracted as an anchor because in MA, “*external granular layer*” is a synonym of MA:*cortical layer II*. On the other hand, there are incorrect ***Type I anchors*** and they mainly come from three cases. (1) Although having the same name, classes in anchor do not represent equivalent entity. For example, MA:*organ system* and NCI:*Organ System*, although sharing matched subclasses, have respective additional different subclasses. (2) Mismatched classes may be considered to be a mapping based on their synonyms or labels. For example, anchor 〈MA:*cerebellum lobule I*, NCI:*Lingula* 〉 (through synonym “*lingula*” in MA) is a mismatch because the former is a part of cerebellar vermis and the latter a part of left lung. (3) Using external resources may introduce incorrect anchors. For example, MA:*back* matches NCI:*Dorsum* because “back” and “dorsum” are synonymous according to the UMLS SPECIALIST Lexicon used in FCA-Map. This is a mismatch because in MA back is a part of trunk, while in NCI dorsum refers to outer surface of scapula.

**Table 9:** Results of lexical anchors

Matching task		Type I	Type II	Total
MA-NCI	Correct	1,164	114	1,278
	Incorrect	60	59	119
	Total	1,224	173	1,397
	Precision	0.951	0.659	0.915
	Recall			0.843
	F-Measure			0.877
FMA-NCI	Correct	2,416	63	2,479
	Unknown	248	4	252
	Incorrect	95	67	162
	Total	2,759	134	2,893
	Precision	0.962	0.485	0.939
	Recall			0.923
	F-Measure			0.931
FMA-SNOMED	Correct	4,563	281	4,844
	Unknown	2,379	98	2,477
	Incorrect	177	186	363
	Total	7,119	565	7,684
	Precision	0.963	0.601	0.930
	Recall			0.804
	F-Measure			0.862
SNOMED-NCI	Correct	10,618	1,076	11,694
	Unknown	725	43	768
	Incorrect	734	565	1,299
	Total	12,077	1,684	13,761
	Precision	0.935	0.656	0.900
	Recall			0.679
	F-Measure			0.774

***Type II*** lexical anchors have lower precisions, reflecting the unstable performance of relying on names sharing tokens to derive commonalities of classes. Nevertheless, many correct ***Type II*** anchors can be identified by the token-based context whereas are missed by other lexical matching methods, as exemplified by 〈MA:*adrenal gland zona reticularis*, NCI:*Reticularis Zone* 〉 and 〈MA:*ileocaecal junction*, NCI:*Ileocecal Valve* 〉. The tokens shared by two classes in such mappings are unique to their names.

### A comparison with TFIDF

**Fig. 8:**
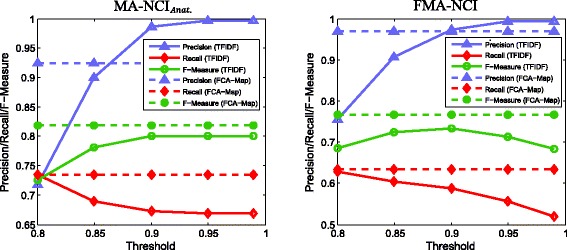
Comparing with TFIDF

Among many lexical matching methods such as string equality, substring test, and edit distance, TFIDF-based methods [1] are of particular interest because similarly to FCA-Map they are based on tokens. Adopted in OM systems YAM++ [5] and GMap [34], TFIDF measures simultaneously how often the tokens occur in one class name and how much information the tokens bring across names of classes from different ontologies. We compare the performance of lexical matching of FCA-Map with TFIDF, solely using the class names of MA and NCI without any external resources. The result is shown in Fig. 8, where F-measure of FCA-Map is higher than TFIDF for any threshold.

Compared with the TFIDF-based methods, FCA-Map emphasizes on the particular commonality of two strings, and there is no need for setting thresholds which is required in TFIDF for selecting matches. This can be illustrated by MA: *tectum* and NCI: *tectum mesencephali*. They are not matched according to TFIDF because token “mesencephali” has a high inverse-document-frequency (it solely occurs in this string) and token “tectum” is ignored (it solely occurs in the two strings). On the other hand, this correspondence can be derived in our method since there is a formal concept with intent {“tectum” } and extent exactly containing these two strings. Moreover, our method can avoid the mistake of locally measuring frequency of tokens. For instance, MA: *common iliac artery* and NCI: *Right Common Iliac Artery* have a relatively high similarity (0.86) according to TFIDF, while this pair is not extracted by FCA-Map. There are many other class names sharing tokens “common”, “iliac”, and “artery”, such as MA: *Left Common Iliac Artery* and NCI: *Right Common Iliac Artery Branch*, therefore what the two strings in comparison share are not unique enough for them to be chosen as a match. Indeed, our method features in detecting the particular commonality solely belongs to the names compared while ignoring the commonality shared by many other names.

In addition to classical TFIDF, there are many other lexical measures, for instance the q-gram based measures [35] and the semantic similarity measures [36]. The former often heavily rely on the threshold used, including variations of TFIDF such as the *ti-idf cosine* measure. The latter are based on the lexical specificity of a class in a large corpus and its category in a semantic hierarchy like WordNet, including the Resnik measure. The experimental comparison with these measures will be for our future work, where biomedical terminologies shall be introduced.

### The results of the relation-based structural validation

Step 2 in FCA-Map constructs the relation-based formal context so as to identify the structural evidence for the lexical anchors, where anchors with negative evidence are eliminated. The results of validated lexical anchors are summarized at the left part of Table 10. One can see that many incorrect ***Type II*** anchors can be eliminated in the validation process, causing the precision to increase in all matching tasks, for instance from 0.659 to 0.778 for ***Type II*** anchors in MA-NCI, and from 0.485 to 0.608 in FMA-NCI. Take ***Type II*** anchor 〈MA:*retina ganglion cell layer*, NCI: *Retinal Ganglion Cell* 〉 for example. It is eliminated by conflict repairing because of its conflict with 〈MA:*retina layer*, NCI: *Retina Layer* 〉, of which the *support degree* is 0 and 8, respectively. The structural validation based on the relation-based concept lattice in FCA-Map can ensure to improve the precision of lexical mappings. This comes with a price though, as shown by the slight decrease of recall when comparing Table 10 with Table 9.

**Table 10:** Results of validated lexical anchors and structural one-to-one mappings

Matching task		Type I	Type II	Total	Structural matches	Total
MA-NCI	Correct	1,161	98	1,259	10	1,269
	Incorrect	59	28	87	5	92
	Total	1,220	126	1,346	15	1,361
	Precision	0.952	0.778	0.935	0.667	0.932
	Recall			0.83		0.837
	F-Measure			0.88		0.882
FMA-NCI	Correct	2,414	48	2,462	2	2,464
	Unknown	208	42	250	0	250
	Incorrect	81	31	112	8	120
	Total	2,703	121	2,824	10	2,834
	Precision	0.968	0.608	0.956	0.20	0.954
	Recall			0.917		0.917
	F-Measure			0.936		0.935
FMA-SNOMED	Correct	4,563	273	4,836	3	4,839
	Unknown	2,379	98	2,477	4	2,481
	Incorrect	177	147	324	5	329
	Total	7,119	518	7,637	12	7,649
	Precision	0.963	0.65	0.937	0.375	0.936
	Recall			0.803		0.803
	F-Measure			0.865		0.865
SNOMED-NCI	Correct	10,618	825	11,443	25	11,468
	Unknown	725	25	750	0	750
	Incorrect	734	304	1,038	39	1,077
	Total	12,077	1,154	13,231	64	13,295
	Precision	0.935	0.731	0.917	0.391	0.914
	Recall			0.665		0.666
	F-Measure			0.771		0.771

### A comparison with the incoherence detection and repairing

The incoherence of mappings refers to the existence of unsatisfiable concepts in the two source ontologies when mappings are introduced, as defined in the ontology validation studies [37]. In these studies, DL reasoners are often used for incoherence detection, i.e., to identify the unsatisfiability, followed by incoherence repairing where mappings are removed so as to regain the satisfiability of the two source ontologies. Conversely in our study, we focus on the conflicts between mappings across ontologies, e.g., in MA, *adipose tissue* is a subclass of *organ system* whereas in NCI, *Adipose Tissue* is disjoint with *Organ System*. Such conflicts may not always cause unsatifiability, though, for instance, when the relation between two mappings is *PART-OF* in one system whereas *DISJOINT-WITH* in the other system.

Despite the distinction, it is worthwhile to conduct an empirical comparison, and we select LogMap [2], a top-ranked OM system that features incoherence diagnosis. Concretely, the repair component of LogMap uses a reasoner, the Dowling-Gallier algorithm [38], to model propositional Horn satisfiability, and employs a greedy strategy to remove mappings with lower weights until satisfiability is recovered. The reasoner, although incomplete for description logics-based ontologies, is highly scalable so that LogMap can process large-scale ontologies in an efficient way. We feed LogMap with the lexical anchors generated from Step 1 of FCA-Map, and the results of the structural validation of FCA-Map (i.e., Step 2) and LogMap are shown in the upper two parts of Table 11. Overall, FCA-Map outperforms LogMap in both recall and F-measure in all of the four matching tasks. The state-of-the-art incoherence repair systems like LogMap tend to heavily rely on the weights of mappings when making choices to remove mappings. FCA-Map, however, does not assign any weights to its mappings, leading LogMap to perform a random removal that may jeopardize the repair quality. On the other hand, as shown by the last rows of the two upper parts of Table 11, for the four matching tasks, the alignment becomes consistent with their source ontologies by LogMap, whereas with FCA-Map the incoherence remains, although the number of unsatisfiable classes is decreased. LogMap pursues coherence of mappings thus favor precision. We further apply LogMap to repair the validated anchors by FCA-Map, and the result is listed in the lower part of Table 11. This combination is of the strictest scrutiny, thus yields the best precision and at the same time the lowest recall in all four tasks in Table 11.

**Table 11:** Comparing the structural validation of FCA-Map with the incoherence repairing of LogMap

Matching task		MA-NCI	FMA-NCI	FMA-SNOMED	SNOMED-NCI
Input mappings		1397	2893	7684	13761
Validated anchors by FCA-Map	N	1346	2824	7637	13231
	P	0.935	0.956	0.937	0.917
	R	0.830	0.917	0.803	0.665
	F	0.880	0.936	0.865	0.771
	Unsat.	14 *↓*11	272 *↓*218	1848 *↓*1836	1352 *↓*947
Repaired anchors by LogMap	N	1385	2709	6415	13129
	P	0.918	0.959	0.952	0.932
	R	0.838	0.859	0.678	0.627
	F	0.876	0.907	0.792	0.750
	Unsat.	14 *↓*0	272 *↓*0	1848 *↓*0	1352 *↓*0
Validated anchors by FCA-Map and then repaired by LogMap	N	1336	2662	6377	12435
	P	0.938	0.967	0.957	0.938
	R	0.827	0.851	0.678	0.619
	F	0.879	0.905	0.793	0.746
	Unsat.	14 *↓*11*↓*0	272 *↓*218*↓*0	1848 *↓*1836*↓*0	1352 *↓*947*↓*0

### The results of the positive relation-based structural matching

Based on the validated lexical anchors, Step 3 of FCA-Map constructs the positive relation-based formal context so as to identify structural mappings. The right part of Table 10 shows the results of additional *one-to-one* mappings and the overall results of the first three steps of FCA-Map. One can see that the quality of such structural one-to-one mappings is limited with low precisions. Nevertheless, as listed in Table 12,^2^, the correct ones are prominent since normally they cannot be identified by lexical methods. As shown by comparing the left and right part of Table 10, these structural mappings, although of small numbers, lead to a slight increase or keep the same in recall in all matching tasks.

**Table 12:** Some one-to-one mappings discovered by the positive relation-based formal contexts

	Mappings
Correct	〈MA:*cerebellar layer*, NCI:*Cortical Cell Layer of the Cerebellum* 〉
	〈MA:*hindlimb bone*, NCI:*Bone of the Lower Extremity* 〉
	〈MA:*arrector pili smooth muscle*, NCI:*Erector Muscle of the Hair* 〉
	〈FMA:*Cardiac muscle tissue*, NCI:*Myocardium* 〉
	〈FMA:*Wall of smooth endoplasmic reticulum*, SNOMED:*Agranular endoplasmic reticulum membrane* 〉
	〈SNOMED:*Forodesine*, NCI:*Immucillin-H* 〉
	〈SNOMED:*Pediculus humanus*, NCI:*Body Lice* 〉
	〈SNOMED:*Structure of metathalamus*, NCI:*Geniculate Body* 〉
	〈SNOMED:*Juvenile neuronal ceroid lipofuscinosis*, NCI:*Batten Disease* 〉
	…
Incorrect	〈MA:*left atrium auricular region*, NCI:*Opening of the Pulmonary Vein* 〉
	〈MA:*septal coronary artery*, NCI:*Left Coronary Artery Branch* 〉
	〈MA:*transverse sinus*, NCI:*Inferior Sagittal Sinus* 〉
	〈FMA:*Amygdala*, NCI:*Cerebral Gray Matter* 〉
	〈SNOMED:*Parakeratosis*, NCI:*Dermatitis* 〉
	〈SNOMED:*Extra embryonic structure*, NCI:*Other Embryologic Structure* 〉
	…
Unknown	〈FMA:*Greater vestibular gland*, SNOMED:*Bartholin s gland structure* 〉
	〈FMA:*Intracranial branch of vertebral artery*, SNOMED:*Cranial branch of vertebral artery* 〉
	…

### Comparing with another structural matching method

In order to evaluate the structural matching of FCA-Map (i.e., Step 3), again we select to compare with LogMap, because its lexical matching and structural matching are separable. Other OM systems are either mainly of lexical analyzing. e.g. AML, or it is impossible or not available to single out a structural matcher. LogMap adopts an on-the-fly unsatisfiability detection and repair mechanism so that the alignment obtained in every iteration of its repair-and-discovery structural matching is always consistent with the two source ontologies. To discover new mappings, LogMap extracts the neighbors of the lexical mappings in the class hierarchy, and computes string similarities of these neighbors across ontologies in order to decide potential matches.

We feed the structural matching of LogMap with the validated lexical anchors generated from Step 2 of FCA-Map, and the results are shown in Table 13. In all of the four matching tasks, LogMap achieves a higher precision due to its rationale of pursuing consistent mappings during the process of structural matching. Nevertheless, FCA-Map is better at recall and finally outperforms LogMap in F-measure for all the four tasks. This is partly due to that FCA-Map exploits more comprehensive structural knowledge in ontology including taxonomy and partonomy whereas LogMap solely uses taxonomical relations. In terms of the pure, structural matches identified and their correctness, as shown by the rightmost columns in the two parts of Table 13, FCA-Map and Log-Map have their respective superiority and inferiority among the four matching tasks.

**Table 13:** Comparing the structural matching of FCA-Map and LogMap

Matching	Input	Structural matching in FCA-Map					Structural matching in LogMap				
Task	mappings	N	P	R	F	Corr./New	N	P	R	F	Corr./New
MA-NCI	1346	1361	0.932	0.837	0.882	10/15	1349	0.934	0.831	0.880	2/8
FMA-NCI	2824	2834	0.954	0.917	0.935	2/10	2696	0.862	0.912	0.907	7/11
FMA-SNOMED	7637	7649	0.936	0.803	0.865	3/12	6596	0.955	0.700	0.808	2/17
SNOMED-NCI	13231	13295	0.914	0.666	0.771	25/64	12248	0.937	0.609	0.738	52/124

### The results of the property matching

The property matching of Step 4 in FCA-Merge is applied to SNOMED-NCI since object properties other than *PART-OF* relationships are solely declared in this matching task, 51 in SNOMED and 82 in NCI. Moreover, there are 29,616 and 6851 equivalent class axioms stated respectively in SNOMED and NCI, providing rich knowledge that enables the corresponding formal contexts to yield mappings across ontologies. Table 14 lists all the property mappings identified between SNOMED and NCI. Both 〈SNOMED:Finding site, NCI:Disease Has Associated Anatomic Site 〉 and 〈SNOMED:Finding site, NCI:Disease Has Primary Anatomic Site 〉 are valid mappings, all describing the sites of diseases while those in NCI are finer-grained than SNOMED. In the next section we will show that such property mappings can facilitate identifying extended and complex correspondences among classes.

**Table 14:** The property mappings identified by the property-based formal context of SNOMED-NCI

SNOMED	NCI
Finding site	Disease has associated anatomic site
Finding site	Disease has primary anatomic site
Due to	Disease has associated disease
Associated morphology	Disease has abnormal cell
Associated morphology	Disease has associated disease
Causative agent	Biological process has result biological process
Has definitional manifestation	Disease has finding

From Table 14, one can see that the number of mappings discovered by the property-based concept lattice is limited. This is partly due to the small proportion of anchors identified and a deficiency of knowledge representation in ontologies. Among the 82 object properties in NCI, 21 describe genes and proteins, such as NCI:Gene Associated With Disease and NCI:Gene Product Encoded By Gene, whereas in SNOMED, there are no properties about genes or proteins. Among the correct anchors, 322 are of genes and proteins, including 〈SNOMED:*Structural gene*, NCI:*Structural Gene* 〉 and 〈SNOMED:*Structural protein*, NCI:*Structural Protein* 〉. 238 of these anchor classes in NCI are linked with one another through relevant properties, as in *Structural Protein*$\sqsubseteq \exists $Gene Product Encoded By Gene.*Structural Gene*. In SNOMED, such axioms do not exist, therefore no mappings can be found for the 21 properties of genes and proteins in NCI.

Moreover, some property mappings can be problematic as exemplified by 〈SNOMED:Due to, NCI:Disease May Have Associated Disease 〉, as extracted from node 3 in Fig. 4. The fillers of object property SNOMED:Due to can be a disease, reaction, event or others, as shown by $ {Falling injury} \sqsubseteq \exists \textsf {Due to}. {Fall} $, whereas the range of property NCI:Disease May Have Associated Disease is defined to be NCI:*Findings and Disorders Kind*. This calls for a manual review by domain experts to decide whether such mappings are valid. Moreover, wrong mappings among classes can induce mismatches of properties. For example, 〈SNOMED:*Hypertensive episode*, NCI:*Hypertensive Episode* 〉 and 〈SNOMED:*Finding of increased blood pressure*, NCI:*Hypertension* 〉 are two anchors used in the property-based formal context for SNOMED-NCI, the former being correct and the latter not. Axioms *Hypertensive episode*$ \sqsubseteq \exists $Has definitional manifestation.*Finding of increased blood pressure* in SNOMED and *Hypertensive Episode*$\sqsubseteq \forall $Disease Has Finding.*Hypertension* in NCI result in the mismatch between SNOMED:Has definitional manifestation and NCI:Disease Has Finding in Table 14.

### The results of the restriction-based structural matching

Applying Step 5 of FCA-Map to constructing the restriction-based formal context is only available for SNOMED-NCI, due to the detection of property mappings. As a result, 394 one-to-one mappings are acquired, 103 of which are correct, causing the recall to increase from 0.666 to 0.672, whereas decreasing the precision from 0.914 to 0.894. Note that the 394 mappings are solely discovered by the restriction-based formal context, some of which are listed in Table 15. Take the correct mapping 〈SNOMED:*Labyrinth structure*, NCI:*Internal Ear* 〉 for example. The two classes share less lexical information so the mapping cannot be obtained from the token-based formal context. Structurally, although the two classes are a subclass of *Ear part* in both SNOMED and NCI, *Ear part* has many other subclasses in the two ontologies so that 〈SNOMED:*Labyrinth structure*, NCI:*Internal Ear* 〉 can not be distinguished. This disables the match to be extracted from the positive relation-based formal context. Finally, the match is detected by the restriction-based formal context built based on the mappings between properties, i.e., from axioms *Sensory hearing loss*$ \sqsubseteq \exists $Finding site.*Labyrinth structure* in SNOMED and *Sensory Hearing Loss*$\sqsubseteq \forall $Disease Has Associated Anatomic Site.*Internal Ear* in NCI.

**Table 15:** Some one-to-one mappings discovered by the restriction-based formal context of SNOMED-NCI

	Mappings
Correct	〈SNOMED:*Labyrinth structure*, NCI:*Internal Ear* 〉
	〈SNOMED:*Structure of lens of eye*, NCI:*Crystalline Lens* 〉
	〈SNOMED:*Structure of gum of maxilla*, NCI:*Upper Gingiva* 〉
	〈SNOMED:*Appendix structure*, NCI:*Vermiform Appendix* 〉
	〈SNOMED:*Structure of cerebral cortex*, NCI:*Cortex* 〉
	…
Incorrect	〈SNOMED:*Tendon sheath structure*, NCI:*Tendon* 〉
	〈SNOMED:*Muscle structure of orbit*, NCI:*Orbit* 〉
	〈SNOMED:*Cheek structure*, NCI:*Buccal Mucosa* 〉
	〈SNOMED:*Cerebellar structure*, NCI:*Vermis* 〉
	〈SNOMED:*Structure of sole of foot*, NCI:*Foot* 〉
	…
Unknown	〈SNOMED:*Parathyroid structure*, NCI:*Parathyroid Gland* 〉
	〈SNOMED:*Upper limb structure*, NCI:*Arm* 〉
	〈SNOMED:*Female mammary gland structure*, NCI:*Female Breast* 〉
	〈SNOMED:*Male mammary gland structure*, NCI:*Male Breast* 〉
	〈SNOMED:*Jaw region structure*, NCI:*Jaw* 〉

On the other hand, the incorrect mappings account for a large proportion, as shown in Table 15, revealing the unstable performance of relying on the restriction-based formal context to derive one-to-one mappings. This is partly due to the granularity difference in knowledge representation between ontologies. About the site of the disease in anchor 〈SNOMED:*Fibroma of tendon sheath*, NCI:*Tendon Sheath Fibroma* 〉, SNOMED is more specific by stating *Fibroma of tendon sheath*$ \sqsubseteq \exists $Finding Site.*Tendon sheath structure* than *Tendon Sheath Fibroma*$ \sqsubseteq \forall $Disease Has Primary Anatomic Site.*Tendon* in NCI. This leads to wrong mapping 〈SNOMED:*Tendon sheath structure*, NCI:*Tendon* 〉. Again, manual reviews from domain experts are necessary to discard the incorrect mappings and retain the correct ones.

All the complex mappings identified from the restriction-based formal context of SNOMED-NCI are listed in Table 16. Note that unlike the one-to-group and group-to-group mappings from the positive relation-based formal context, classes within a complex mapping in Table 16 are of different types, e.g., SNOMED:*Vascular structure of liver* and NCI:*Blood Vessel* are of the same type whereas they and NCI:*Liver* represent different things. Thus the semantic expressions in Table 16 are no longer mere disjunctions of classes, and manual reviews decide what properties and logical constructors shall be used to impose restrictions on classes.

**Table 16:** The complex mappings discovered by the restriction-based formal context of SNOMED-NCI

	Classes	Semantic expression
SNOMED	Vascular structure of liver	Vascular structure of liver
NCI	Liver, Blood Vessel	Blood Vessel ⊓ ∃*PartOf*.Liver
SNOMED	Vascular structure of penis	Vascular structure of penis
NCI	Penis, Blood Vessel	Blood Vessel ⊓ ∃*PartOf*.Penis
SNOMED	Blood vessel structure of skin	Blood vessel structure of skin
NCI	Skin, Blood Vessel	Blood Vessel ⊓ ∃*PartOf*.Skin
SNOMED	Abdominal vascular structure	Abdominal vascular structure
NCI	Abdominal Cavity, Blood Vessel	Blood Vessel ⊓ ∃*PartOf*.Abdominal Cavity
SNOMED	Structure of soft tissues of head and neck	Structure of soft tissues of head and neck
NCI	Head and Neck, Connective and Soft Tissue	Connective and Soft Tissue ⊓ ∃*PartOf*.Head and Neck
SNOMED	Structure of soft tissues of head	Structure of soft tissues of head
NCI	Head, Connective and Soft Tissue	Connective and Soft Tissue ⊓ ∃*PartOf*.Head
SNOMED	Structure of soft tissues of neck	Structure of soft tissues of neck
NCI	Neck, Connective and Soft Tissue	Connective and Soft Tissue ⊓ ∃*PartOf*.Neck
SNOMED	Structure of submandibular lymph node	Structure of submandibular lymph node
NCI	Submandibular Gland, Lymph Node	Lymph Node ⊓ ∃*PartOf*.Submandibular Gland
SNOMED	Structure of lymph node of mesentery	Structure of lymph node of mesentery
NCI	Mesentery, Lymph Node	Lymph Node ⊓ ∃*PartOf*.Mesentery
SNOMED	Skin structure of scrotum	Skin structure of scrotum
NCI	Scrotum, Skin	Skin ⊓ ∃*PartOf*.Scrotum
SNOMED	Skin structure of breast	Skin structure of breast
NCI	Breast, Skin	Skin ⊓ ∃*PartOf*.Breast
SNOMED	Skin structure of ear	Skin structure of ear
NCI	Ear, Skin	Skin ⊓ ∃*PartOf*.Ear

In order to evaluate the correctness of the complex mappings, we feed them into the repair component of LogMap which calls a reasoner to check the satisfiability of the alignment integrated with two source ontologies. Concretely, the semantic expressions as shown in Tables 16 and 5 are transformed into equivalent class axioms, which are 43 for MA-NCI, 7 for FMA-NCI, 30 for FMA-SNOMED, and 75 for SNOMED-NCI, 12 being in the form of restrictions from Table 16 and the others all disjunctions of classes as in Table 5. For example, based on the first line in Table 16, we generate an equivalent axiom in NCI, *C*_*NCI*_≡*BloodVessel*⊓ ∃*PartOf*.*Liver* where *C*_*NCI*_ is an artificial class, and we pair *C*_*NCI*_ and SNOMEDT class *Vascular structure of liver* as a mapping. LogMap reports coherence for three alignments, FMA-NCI, FMA-SNOMED and SNOMED-NCI, whereas for MA-NCI, LogMap detects two unsatisfiable classes in NCI. Complex mappings lead NCI classes like *Bronchial Secretion* and *Cardiovascular System* to become equivalent, and the former is a subclass of *Body Fluid or Substance* while the latter of *Organ System*, which are declared to be disjoint in NCI.

### A comparison with the OAEI 2016 top-ranked systems

We compare the performance of the first three steps of FCA-Map with the OAEI 2016 top-ranked systems, XMap [3], AML [4], LogMap [2], and LogMapBio [39], on all matching tasks in the OAEI 2016 three biomedical tracks and the Conference track. In addition to small fragments as in Table 2, the Large Biomedical Ontologies track contains matching tasks for the whole FMA and NCI, and a larger proportion of SNOMED with up to 120 thousand classes, as listed in the upper part of Table 17. The Disease and Phenotype track [31] is organized by Pistoia Alliance Ontologies Mapping project team based on a real use case for finding alignments between disease and phenotype ontologies. Specifically, the selected ontologies are the Human Phenotype Ontology (HP), the Mammalian Phenotype Ontology (MP), the Human Disease Ontology (DOID), and the Orphanet and Rare Diseases Ontology (ORDO), for which four matching tasks are designed, shown by the lower part of Table 17. Moreover, the Conference track [31] consists of 16 ontologies about different conference organizations. These ontologies are of small scale, with classes from 14 to 140 and object properties from 13 to 61.

**Table 17:** Matching tasks of whole ontologies in the OAEI 2016 Large Biomedical Ontologies track and the Disease and Phenotype track

Matching task	Number of classes in *O*_1_	Number of classes in *O*_2_
FMA-NCI Whole	78,989	66,724
FMA-SNOMED Whole	78,989	122,464 (40% of SNOMED)
SNOMED-NCI Whole	122,464 (40% of SNOMED)	66,724
HP-MP vote 2, vote 3	11,828	11,752
DOID-ORDO vote 2, vote 3	9,301	12,974

The results are shown in Table 18, as officially reported by OAEI [31]. In the Anatomy track, the precision, recall and F-measure of FCA-Map for MA-NCI ranks second, fifth, and forth, respectively. Results on the Large Biomedical Ontology track are more encouraging, where FCA-Map ranks second for both F-measures of FMA-NCI and FMA-SNOMED, and ties for third for F-measure of SNOMED-NCI. More strikingly, for SNOMED-NCI Whole, the largest ontology matching task in OAEI, FCA-Map ranks first for recall and second for F-measure. For other two tasks of the whole ontologies, the recall of FCA-Map ranks for second, whereas its performance on precision is unsatisfactory.

**Table 18:** Comparing FCA-Map with the OAEI 2016 top-ranked systems

Matching task		XMap	AML	LogMap	LogMapBio	FCA-Map
Conference	Precision	0.85	0.84	0.82	0.77	0.75
	Recall	0.57	0.66	0.59	0.56	0.52
	F-Measure	0.68	0.74	0.69	0.65	0.61
MA-NCI	Precision	0.929	0.95	0.918	0.888	0.932
	Recall	0.865	0.936	0.846	0.896	0.837
	F-Measure	0.896	0.943	0.88	0.892	0.882
FMA-NCI	Precision	0.977	0.936	0.949	0.935	0.954
	Recall	0.901	0.902	0.901	0.910	0.917
	F-Measure	0.937	0.931	0.924	0.923	0.935
FMA-SNOMED	Precision	0.989	0.953	0.948	0.944	0.936
	Recall	0.846	0.727	0.690	0.696	0.803
	F-Measure	0.912	0.825	0.799	0.801	0.865
SNOMED-NCI	Precision	0.911	0.904	0.922	0.896	0.914
	Recall	0.564	0.713	0.663	0.675	0.666
	F-Measure	0.697	0.797	0.771	0.770	0.771
FMA-NCI Whole	Precision	0.902	0.838	0.854	0.818	0.409
	Recall	0.847	0.872	0.802	0.835	0.872
	F-Measure	0.874	0.855	0.827	0.826	0.557
FMA-SNOMED Whole	Precision	0.965	0.882	0.839	0.808	0.452
	Recall	0.843	0.687	0.634	0.640	0.773
	F-Measure	0.900	0.773	0.722	0.714	0.571
SNOMED-NCI Whole	Precision	−	0.904	0.870	0.842	0.786
	Recall	−	0.668	0.596	0.637	0.686
	F-Measure	−	0.768	0.708	0.725	0.732
HP-MP vote 2	Precision	1.000	0.931	0.935	0.918	0.984
	Recall	0.333	0.800	0.913	0.932	0.754
	F-Measure	0.500	0.860	0.924	0.925	0.854
HP-MP vote 3	Precision	1.000	0.854	0.773	0.755	0.942
	Recall	0.435	0.945	0.973	0.982	0.924
	F-Measure	0.606	0.897	0.862	0.854	0.933
DOID-ORDO vote 2	Precision	0.985	0.853	0.952	0.920	0.966
	Recall	0.569	0.971	0.878	0.898	0.959
	F-Measure	0.721	0.908	0.913	0.909	0.962
DOID-ORDO vote 3	Precision	0.977	0.778	0.905	0.864	0.888
	Recall	0.632	0.998	0.938	0.949	0.993
	F-Measure	0.767	0.878	0.921	0.905	0.937

In the Disease and Phenotype track [40], note that there are none reference mappings; instead, consensus alignments representing the agreements of the participating OM systems are used for evaluation. Out of the four tasks, FCA-Map produces the closest results to the consensus alignments in terms of F-measure in three tasks, and the second close results in terms of precision in three tasks. Compared with the large biomedical ontologies, in all the ontologies of the track, HP, MP, DOID and ORDO, there are none disjoint axioms declared. This may to some extent affect the satisfiability checking-based mapping diagnosis in OM systems like LogMap and LogMapBio. In FCA-Map, on the other hand, the structural validation of Step 2 largely increases the precision by eliminating several hundreds of lexical anchors in all four matching tasks. The benefit comes from its anchor screening operation which identifies the *Type II lexical anchors* without any structural evidence as mismatches. Such structural isolations may be partly due to the absence of partonomic relations, as neither HP nor MP declares any PART-OF property, and in DOID there are only six uses of the PART-OF relationship.

All these results indicates that FCA-Map can achieve a better balance between precision and recall for biomedical ontologies, through incrementally constructing multiple FCA structures to detect and validate various kinds of mappings. In contrast, in the Conference track, as shown by the average values in the first row of Table 18, FCA-Map comes last in all three measures. The Conference ontologies are of smaller sizes, leading to smaller-sized formal contexts in FCA-Map, from which the derivation of commonalities among classes becomes ineffective.

In terms of the runtime, OAEI regulates that OM systems fail to finish a matching task within two hours are not considered in the evaluations. Among the tasks listed in Table 18, FCA-Map is thus not reported by OAEI on SNOMED-NCI and all three whole ontology tasks in the Large Biomedical Ontologies track. Every step of FCA-Map is composed of three subsequent parts, constructing a formal context, deriving a concept lattice, and extracting mappings. Among them, the derivation of a formal concept lattice of FCA is of high complexity as a PSPACE-complete problem, and the number of formal concepts in a lattice can be exponential with the size of the formal context. This means that every step of FCA-Map is computationally complex, and Step 1 generally takes a longer time than other steps since the token-based formal context tends to be larger. Moreover, the richer lexical and structural knowledge described in the ontologies, the larger the formal contexts constructed, leading the lattices to grow significantly. To optimize, we multithread the code for lattice computation which results in a great deal of saving of time. Table 19 shows the runtimes of FCA-Map on the OAEI biomedical ontologies in our own running setup. Completing the SNOMED-NCI task becomes available within two hours now (was 3.5 h), and the time for three whole ontology matching tasks has dropped from 20, 25, and 30 h to 7, 8 and 13 h, respectively.

**Table 19:** Runtimes of the steps in FCA-Map

Matching task	Running time (seconds)			
	Step 1	Step 2	Step 3	Total
MA-NCI	15	9	7	33
FMA-NCI	69	35	23	130
FMA-SNOMED	251	538	431	1226 (0.34 h)
SNOMED-NCI	2262	2590	1890	6759 (1.88 h)
FMA-NCI Whole	23764	739	362	24877 (6.9 h)
FMA-SNOMED Whole	21225	5261	3728	30240 (8.4 h)
SNOMED-NCI Whole	36212	6747	4605	47599 (13.2 h)
HP-MP vote 2, vote 3	1262	2	2	1270 (0.35 h)
DOID-ORDO vote 2, vote 3	825	2	2	837 (0.23 h)

### A comparison with FCA-Merge

The previous FCA-based OM systems have not participated in OAEI, whereas it is worth conducting an empirical comparison with them. Particularly, we select FCA-Merge [15], the renowed ontology matching system that innovatively exploited FCA. In the formal context, FCA-Merge uses textual documents crawled from the Web as objects, and classes in two source ontologies as attributes. The domain that FCA-Merge demonstrates is tourism, and the cell in the formal context is marked if the corresponding class’s name occurs in the touring article. The code of FCA-Merge is not available, so we follow its way to construct formal contexts in the domain of biomedicine. Concretely, the PubMed articles [41] are used as objects and classes in two source biomedical ontologies from Table 2 as attributes. The binary relation holds if the class in the column has its name, label, or synonym occur in the abstract of the PubMed article in the row. The PubMed is the largest repository of biomedical literature, and we focus on articles about clinical medicine and translational medicine. As a result, 24907 articles are retrieved. Both their abstracts and the names/labels/synonyms of ontology classes are normalized by the UMLS SPECIALIST Lexicon [29] where ordering and stop words removal are not applied.

Based on the heuristics that FCA-Merge employs to merge classes, in the lattice derived, if the simplified intent of a formal concept contains one class from one ontology and *n* classes from another ontology, *n* one-to-one mappings are extracted. The results are shown in Table 20, where one can see that FCA-Merge favors precision significantly over recall, the former all being higher than 0.9 whereas the latter lower than 0.3. Compared with the first three steps of FCA-Map, FCA-Merge performs better in precision only for MA-NCI, and all its recalls and F-measures are uncompetitive. Nevertheless, as shown by the right column of Table 20, FCA-Merge has successfully identified a few correct mappings that are missed by FCA-Map, for instance, 〈FMA:*Stroma*, NCI:*Stroma Connective Tissue* 〉 which occur in the simplified intent of a formal concept with 403 articles as extent. Moreover, the effectiveness of FCA-Merge obviously depends on the collection of textual documents used, and expanding the PubMed articles in the formal context may improve the recall.

**Table 20:** Comparing FCA-Map with FCA-Merge

Matching task	FCA-Map				FCA-Merge				
	N	P	R	F	N	P	R	F	Additional mappings vs FCA-Map
MA-NCI	1361	0.932	0.837	0.882	425	0.939	0.263	0.411	16 (0 correct)
FMA-NCI	2834	0.954	0.917	0.935	834	0.946	0.294	0.488	42 (6 correct)
FMA-SNOMED	7649	0.936	0.803	0.865	373	0.909	0.056	0.106	32 (3 correct)
SNOMED-NCI	13295	0.914	0.666	0.771	3051	0.878	0.156	0.265	328 (38 correct)

## Discussion

FCA-Map distinguishes in comprehensively exploiting the FCA formalism in matching real-world biomedical ontologies. In this section, by introducing some of the OAEI top-ranked systems, the previous FCA-based systems and the systems capable of identifying complex mappings, we analyze what FCA-Map has in common with them and its distinctive features. The limitations and thus our future work are also discussed in detail.

### Comparing with the OAEI 2016 top-ranked systems

Among the OAEI 2016 top-ranked participants, LogMap [2] is a scalable OM system that uses lexical and semantic indexing techniques, and when dealing with mapping incoherence, it runs a reasoning-based diagnosis and inconsistency repairing. AgreementMakerLight (AML) [4] is an automated OM system that primarily uses the element-level techniques. XMap [3] is an automated OM system that composes various kinds of basic ontology matchers. Both AML and XMap uses external resources as background knowledge.

FCA-Map shares with these OM systems in exploiting lexical and structural knowledge in ontology to detect correspondences among classes across ontologies. Moreover, external domain resources like UMLS are used to facilitate the alignment. Compared with these systems, FCA-Map is distinctive in relying on a mathematical model to compute commonalities among classes and properties. As a result, FCA-Map is capable of obtaining mappings that cannot be identified by other systems, as exemplified by anchors 〈MA:*adrenal gland zona reticularis*, NCI:*Reticularis Zone* 〉 and 〈MA:*ileocaecal junction*, NCI:*Ileocecal Valve* 〉. These mappings are identified in the token-based concept lattice and then validated in the relation-based concept lattice. The tokens shared by two classes in these mappings are unique to their names. The lexical matching method of FCA-Map is suitable for domain ontologies having class names, labels, or synonyms from domain-specific vocabularies. Other concept lattices derived in FCA-Map can also detect mappings that are absent in other OM systems, e.g., 〈SNOMED:*Deoxyribonucleic acid virus*, NCI:*DNA Virus* 〉 and 〈SNOMED:*Jobs syndrome*, NCI:*Hyperimmunoglobulin E Syndrome* 〉 from the positive relation-based lattice, and 〈SNOMED:*Structure of gum of maxilla*, NCI:*Upper Gingiva* 〉 and 〈SNOMED:*Intrahepatic biliary tract structure*, NCI:*Intrahepatic Bile Duct* 〉 from the restriction-based lattice.

Table 21 lists the mappings that are uniquely identified by FCA-Map compared with all OAEI 2016 participants. For all the seven matching tasks in the Anatomy and Large Biomedical Ontologies track, FCA-Map managed to discover correct mappings for which the corresponding OAEI systems failed. Take SNOMED and NCI for example. For their fragment matching task, 2286 mappings identified by FCA-Map are absent from all the five participants of the task, 1175 of which are correct; and for their whole matching task, 2743 FCA-Map mappings are missed by all the four participants, where 503 are correct.

**Table 21:** Unique mappings identified by FCA-Map compared with all OAEI 2016 participants

Matching task	Reference alignment	FCA-Map				OAEI 2016 Participants
		N	P	R	Uniquely identified mappings	
MA-NCI	1516	1361	0.932	0.837	34 (6 correct)	12 (Alin, AML, CroMatcher, DKP _*AOM*_Lite, DKP _AOM, Lily, LogMap, LogMapBio, LogMapLite, LPHOM LYAM, XMap)
FMA-NCI	3024	2834	0.954	0.917	75 (8 correct)	10 (Alin, AML, DKP _*AOM*_Lite, DKP _AOM, Lily, LogMap, LogMapBio, LogMapLite LYAM, XMap)
FMA-NCI Whole	3024	6449	0.386	0.892	3038 (28 correct)	5 (AML, LogMap, LogMapBio, LogMapLite, XMap)
FMA-SNOMED	9008	7649	0.936	0.803	669 (115 correct)	5 (AML, LogMap, LogMapBio, LogMapLite, XMap)
FMA-SNOMED Whole	9008	15391	0.352	0.759	8542 (104 correct)	5 (AML, LogMap, LogMapBio, LogMapLite, XMap)
SNOMED-NCI	18844	13295	0.914	0.666	2286 (1175 correct)	5 (AML, LogMap, LogMapBio, LogMapLite, XMap)
SNOMED-NCI Whole	18844	16452	0.775	0.705	2743 (503 correct)	4 (AML, LogMap, LogMapBio, LogMapLite)

Nevertheless, in terms of the evaluation measures, FCA-Map, although competitive on some tasks, does not outperform the OAEI top-ranked systems. These systems often integrate various, distinctive matchers, including combing many lexical metrics (e.g., in the case of AML), as well as statistical and machine-learning algorithms. Better performances can be achieved through the complementation of diverse matchers. As shown in [42], optimal string similarity metrics alone can produce competitive results with the state-of-the-art OM systems. Conversely, the goal of our study, rather than optimally selecting a group of different algorithms, is to investigate how one, single mathematical formalism can be empowered to facilitate ontology matching based on its feature of clustering attribute commonalities among objects. To improve FCA-Map for practical applications, other matching techniques can be incorporated and the integration strategies shall be studied.

### Comparing with previous FCA-based OM systems

Among the previous FCA-based OM systems, FCA-Merge [15] proposed by Stumme and Maedche extracts instances for classes from domain documents based on the natural language processing technology and constructs two formal contexts for the ontologies to be merged, respectively. A common context is computed, and a concept lattice derived from which the final merged ontology can be generated. FCA-OntMerge [23] uses the classes of ontologies as objects in its formal context, and applies strict mathematical principles of the concept lattice derived to guiding the merge of two ontologies. In [22], again ontology classes are used as objects in the formal context, whereas the attributes come from the terms of a domain-specific thesaurus, so that similarity measures can be computed for identifying class mappings. Further, alternative FCA structures are adopted in ontology mapping including the fuzzy formal concept analysis (FFCA) formalism. Take FFCA-Merge [24] for example, which extends FCA-Merge to generate fuzzy ontologies by combining WordNet and FFCA. The work of [22] is also extended by FFCA in [26] and by the rough set theory in [25], respectively, in order to handle the uncertain information among different ontologies.

Compared with these previous FCA-based OM systems, FCA-Map is more comprehensive in utilizing as much knowledge represented in ontologies as possible. Table 22 lists the components used as objects and attributes when building formal contexts in the FCA-based OM systems. The lexical information used in FCA-Map includes names of classes as well as their labels and synonyms when available, augmented by domain-specific terminologies and lexical tools. The structural knowledge used in FCA-Map includes *ISA* and *PART-OF* hierarchies, disjointed and sibling relations among classes, axioms between named classes, and axioms between a named class and restrictions about object properties. More importantly, both asserted and inferred axioms are exploited. With these knowledge, five types of FCA formal contexts are constructed in an incremental way, and from the concept lattices derived mappings can be extracted and validated automatically. As demonstrated by the evaluation on large, complex biomedical ontologies, FCA-Map can achieve a better balance between precision and recall through multiple, incremental FCA structures and a combination of detection and validation operations. Specifically, the lexical matching of Step 1 in FCA-Map is devised to pursue both precision and recall; the validation of Step 2 eliminates mappings with negative structural evidence and ensures the improvement of precision; and the structural matching in Step 3, 4 and 5 discover new, structural matches to favor recall. As a result, for SNOMED-NCI Whole, the largest ontology matching task in OAEI, FCA-Map ranks first for recall and second for F-measure; ranks second for both F-measures of FMA-NCI and FMA-SNOMED, and obtains the best F-measures for most Disease and Phenotype tasks.

**Table 22:** FCA-based ontology merging and matching systems

FCA-based systems for ontology merging or matching		Object in the formal context	Attribute in the formal context	Binary relation in the formal context
FCA-Merge, FFCA-Merge (merging)		Textual document	Class from two ontologies	Name of class occurs in document
[22*,*^25^*,*^26^] (matching)		Ontology class	Thesaurus term	Term occurs in class
FCA-OntMerge (merging)		Class from two ontologies	Attribute from two ontologies	Class has attribute
FCA-Map (matching)	Step 1: lexical matching	Name, label, or synonym of classes from two ontologies	Token	String contains token
	Step 2: structural validation	Class from two ontologies	Lexical anchor prefixed with a relation	Class has relation with anchor
	Step 3: structural matching	Class from two ontologies	Validated anchor prefixed with a relation	Class has relation with validated anchor
	Step 4: property matching	Property from two ontologies	A pair of anchors	Property links the individuals of two anchors
	Step 5: extended structural matching	Class from one ontology	Class from another ontology	Two classes across ontologies occur in the anonymous ancestors of the same anchor with the same property

The FCA-Map method is suitable for aligning large-scale domain ontologies with rich lexical and structural knowledge. As shown by the SNOMED-NCI matching task in the evaluation, both ontologies declare object properties and specify equivalent class axioms using the properties. This provides semantic linkage rich enough for the property-based and restriction-based formal contexts to yield mappings across ontologies. Moreover, the large number of classes and their semantic relations enable the derivation of commonalities among classes. On the other hand, the performance of FCA-Map can be relatively poor for smaller-sized and general-purpose ontologies whose terminologies are more varied and ambiguous, like those in the Conference track of OAEI.

### Identifying complex mappings

In addition to one-to-one class matches, FCA-Map can identify meaningful complex mappings and property mappings. Complex mappings are those in contrast with one-to-one mappings across ontologies, and there have been many OM systems that are capable of identifying varying kinds of complex correspondences, e.g., as listed in [1], iMAP, DCM, HSM, AOAS, PORSCHE, AML, and Optima. These works mostly use predefined mapping structures as a guidance to identify complex mappings, e.g., manually created complex structures in AOAS and logical definitions of the ontologies per se in AML. FCA-Map, on the other hand, benefits from the formal concept formalism of FCA, which clusters commonalities among a group of entities rather than solely two, a natural way to revealing complex correspondences across ontologies. This means that no extra runtimes are needed for identifying complex mappings in FCA-Map. Note that FCA-Map is not the only system independent of predefined complex structures. DCM, for example, takes co-occurrence patterns as suggestive complex matches, where a large-sized data is required for mining such patterns. Additionally, complex mappings combing multiple classes and properties across ontologies may indicate the absence of a class representing that complex semantic meaning within ontology, thus can be used for quality assurance of large, real-world biomedical ontologies [43].

In contrast with classes, property aligning among ontologies is less studied. As shown by [42], string similarity metrics perform significantly poor on properties, indicating the unreliability of lexical-based property matching. FCA-Map, independent of the names of properties, particularly devises a formal context to describe how properties have commonalities in connecting the same classes across ontologies. In our evaluation, although of a small number, the property mappings identified between SNOMED and NCI facilitate detecting extended one-to-one and complex class mappings. Lastly, for complex mappings and object mappings, manual reviews are required, not only for detecting the mismatches, but also for clarifying different semantic relations and logical connections among classes in the mapping.

### Limitations and future work

From the experimental results, one can see that as the step-by-step process of FCA-Map goes more relying on the structures of ontologies, the mapping results become more unreliable. This is understandable, as ontologies for the same domain tend to differ structurally while agree more on names. Practically, manual validation from domain experts is necessary at each step so that the mismatches do not propagate further. Technically, the structural validation of Step 2 in FCA-Map should be performed whenever new matches are added, i.e., after Step 3, 4, and 5, so that the conflicting semantic relations among mappings can be eliminated. This would for sure increase the correctness of structural matching. More importantly, FCA-Map lacks the incoherence detection and repairing, an indispensable part of ontology matching, resulting in many unsatisfiable classes as reported by OAEI 2016. Without them the correctness of structural matching could be further improved. Note that incoherence repairing methods relying on the weights to decide the mappings to be removed are not suitable for FCA-Map as its mappings are not weighted. Whether it is possible to equip FCA per se to accomplish incoherence repairing will be for our future work. Conversely, alternatives to improve the recall are also worth exploring. Starting with a more relaxed first step in FCA-Map could lead the lexical anchors to have much higher recall and lower precision. For instance, when the class-origin extent of the token-based formal concept contains *three* classes across ontologies, we can extract three one-to-one mappings from them. Such extractions will be noisier than the current Step 1 of FCA-Map, for which both the structural validation and incoherence repairing are required to ensure the quality of the final alignment. Moreover, property mappings identified in Step 4 can enable a new round of structural matching so that in addition to taxonomic and partonomic relations, equivalent properties across ontologies can be used to augment the positive relation-based formal context.

Another limitation that FCA-Map suffers is the long running time. As a PSPACE-complete problem, computing a concept lattice of FCA can be space- and time-consuming. By taking advantage of the existing FCA tool FCAlib that computes the polynomial-sized Galois Sub-hierarchy (GSH) representation of concept lattice, and applying the multithreading technique, we managed to finish the Anatomy, the Disease and Phenotype, and the fragment tasks of the Large Biomedical Ontologies track of OAEI 2016 within two hours as required. However, when it comes to the whole versions of the Large Biomedical Ontologies track, FCA-Map took an average of nearly 10 h. For OM tasks of such size and complexity, Spark-MCA [44], a newly developed technology for tackling the computational challenge of large biomedical ontologies based on distributed cloud computing frameworks, can be considered as a solution.

Additionally, an interesting direction for our future work would be to exploit the formal concept analysis formalism to align multiple ontologies at the same time. Preliminarily, we applied FCA-Map to constructing a token-based formal context for three ontologies, FMA, NCI and SNOMED in Table 2. The names, labels and synonyms of classes in the three ontologies are listed as the objects of the context, and the tokens in these strings as the attributes. After the lattice derivation, one-to-one lexical mappings are extracted from the formal concepts by Step 1 of FCA-Map. Indirect mappings can then be generated from two mappings sharing a class. Take matching task SNOMED-NCI for example. Using FMA as an intermediate yielded 779 mappings where 246 are solely of indirect matching. Among these unique mappings, 165 are correct, increasing both the recall and F-measure whereas the precision is lowered. In-depth analyses on adapting FCA-Map to aligning multiple ontologies are needed.

## Conclusion

To conclude, the study in this paper attempts to push the envelope of the Formal Concept Analysis formalism in ontology matching tasks. In our system FCA-Map, five types of formal contexts are constructed in an incremental way, and their derived concept lattices are used to cluster the commonalities among classes and properties at various lexical and structural levels, respectively. Experiments on large, real-world biomedical ontologies show promising results and reveal the power of FCA. Relying on one, single formalism, the performance of FCA-Map is competitive with the OAEI top-ranked participants, and it can uniquely identify mappings that are missed by other OM systems. Additionally, complex mappings are obtained at the same time as one-to-one mappings in FCA-Map, indicating that one class corresponds to a semantic expression in the other ontology. Compared with previous FCA-based OM systems that normally constructs one formal context, our method features a comprehensive incorporation of various kinds of knowledge in ontology into multiple FCA contexts, including class names, labels, and synonyms, and taxonomy, partonomy, and axioms restricting properties among classes. FCA-Map can thus be applied to aligning domain ontologies with such knowledge richly represented. To further our study, extensions with incoherence repairing and optimization techniques are definitely worth exploring so as to improve the quality and efficiency of ontology matching with FCA-Map.

## Abbreviations

AML: AgreementMakerLight
FCA: Formal concept analysis
FFCA: Fuzzy formal concept analysis
FMA: Foundational model of anatomy
GSH: Galois sub-hierarchy
MA: Adult mouse anatomy
NCI: National cancer institute thesaurus
OAEI: Ontology Alignment Evaluation Initiative
OM: Ontology matching
SNOMED-CT: Systematized nomenclature of medicine – clinical terms
UMLS: Unified medical language system

## Footnotes

1 Although eliminating the stop words carrying logical meanings may affect the precision, its benefit in recall is more advantageous according to our experiments.

2 Again, “Unknown” mappings, although from the UMLS metathesaurus, are categorized by OAEI as neither correct nor incorrect.

## References

[1] Euzenat J, Shvaiko P (2013). Ontology Matching. 2nd ed..

[2] Jiménez-Ruiz E, Grau BC (2011). Logmap: Logic-based and scalable ontology matching. International Semantic Web Conference.

[3] Djeddi WE, Khadir MT (2010). Xmap: a novel structural approach for alignment of owl-full ontologies. Machine and Web Intelligence (ICMWI), 2010 International Conference On.

[4] Faria D, Pesquita C, Santos E, Palmonari M, Cruz IF, Couto FM (2013). The agreementmakerlight ontology matching system. OTM Confederated International Conferences" On the Move to Meaningful Internet Systems".

[5] Duyhoa N, Bellahsene Z (2012). Yam++ results for oaei 2012. Seventh International Workshop on Ontology Matching.

[6] Niepert M, Meilicke C, Stuckenschmidt H (2010). A Probabilistic-Logical Framework for Ontology Matching. Proceedings of the Twenty-Fourth AAAI Conference on Artificial Intelligence.

[7] Diallo G. (2014). An effective method of large scale ontology matching. J Biomed Semant.

[8] Gulić M, Vrdoljak B, Banek M (2016). Cromatcher: An ontology matching system based on automated weighted aggregation and iterative final alignment. Web Semant Sci Serv Agents World Wide Web.

[9] Foundational Model of Anatomy. http://si.washington.edu/projects/fma. Accessed 21 Sept 2017.

[10] Adult Mouse Anatomy. http://www.informatics.jax.org/glossary/adult_ma_dictionary. Accessed 21 Sept 2017.

[11] National Cancer Institute Thesaurus. https://ncit.nci.nih.gov/. Accessed 21 Sept 2017.

[12] Systematized Nomenclature of Medicine – Clinical Terms. http://www.snomed.org/snomed-ct/. Accessed 21 Sept 2017.

[13] Lindberg DA, Humphreys BL, McCray AT (1993). The unified medical language system. IMIA Yearbook.

[14] Evaluation Alignment Initiative. http://oaei.ontologymatching.org/. Accessed 21 Sept 2017.

[15] Stumme G, Maedche A (2001). Fca-merge: Bottom-up merging of ontologies. IJCAI.

[16] Wille R (1982). Restructuring lattice theory: an approach based on hierarchies of concepts. Ordered Sets.

[17] Uschold M, Gruninger M (1996). Ontologies: Principles, methods and applications. Knowl Eng Rev.

[18] Cimiano P, Hotho A, Stumme G, Tane J (2004). Conceptual knowledge processing with formal concept analysis and ontologies. International Conference on Formal Concept Analysis.

[19] Bain M (2003). Inductive construction of ontologies from formal concept analysis. Australasian Joint Conference on Artificial Intelligence.

[20] Bendaoud R, Napoli A, Toussaint Y (2008). Formal concept analysis: A unified framework for building and refining ontologies. International Conference on Knowledge Engineering and Knowledge Management.

[21] Obitko M, Snásel V, Smid J (2004). Ontology design with formal concept analysis. CLA.

[22] de Souza KXS, Davis J (2004). Aligning ontologies and evaluating concept similarities. OTM Confederated International Conferences" On the Move to Meaningful Internet Systems".

[23] Guan-yu L, Shu-peng L (2010). Formal concept analysis based ontology merging method. Computer Science and Information Technology (ICCSIT), 2010 3rd IEEE International Conference On.

[24] Chen R-C, Bau C-T, Yeh C-J (2011). Merging domain ontologies based on the wordnet system and fuzzy formal concept analysis techniques. Appl Soft Comput.

[25] Zhao Y, Wang X, Halang W (2006). Ontology mapping based on rough formal concept analysis. Advanced Int’l Conference on Telecommunications and Int’l Conference on Internet and Web Applications and Services (AICT-ICIW’06).

[26] Xu X, Wu Y, Chen J (2010). Fuzzy fca based ontology mapping. 2010 First International Conference on Networking and Distributed Computing.

[27] Ganter B, Wille R (2012). Formal Concept Analysis: Mathematical Foundations.

[28] Sub-Term Mapping Tools. https://lsg2.nlm.nih.gov/LexSysGroup/Projects/stmt/2013+/web/index.html. Accessed 04 Sept 2017.

[29] UMLS SPECIALIST Lexicon. https://lexsrv3.nlm.nih.gov/LexSysGroup/Projects/lexicon/current/web/index.html. Accessed 04 Sept 2017.

[30] Zhang S, Bodenreider O (2007). Experience in aligning anatomical ontologies. Int J Semant Web Inf Syst.

[31] Achichi M, Cheatham M, Dragisic Z, Euzenat J, Faria D, Ferrara A, Flouris G, Fundulaki I, Harrow I, Ivanova V (2016). Results of the ontology alignment evaluation initiative 2016. CEUR Workshop Proceedings, vol. 1766.

[32] Godin R, Mili H (1993). Building and maintaining analysis-level class hierarchies using galois lattices. ACM SIGplan Notices, vol. 28.

[33] FCAlib. https://julianmendez.github.io/fcalib/. Accessed 21 Sept 2017.

[34] Li W. Combining sum-product network and noisy-or model for ontology matching. Germany: CEUR-WS RWTH Aachen University; 2015. pp. 35–9.

[35] Hassanzadeh O, Sadoghi M, Miller RJ. Accuracy of Approximate String Joins Using Grams. In: Proceedings of the Fifth International Workshop on Quality in Databases, QDB 2007, at the VLDB 2007 conference, Vienna, Austria, September 23, 2007: 2007. p. 11–8. http://dblp.org/rec/bib/conf/iqis/HassanzadehSM07.

[36] Patwardhan S, Banerjee S, Pedersen T (2003). Using measures of semantic relatedness for word sense disambiguation. Proceedings of the 4th International Conference on Computational Linguistics and Intelligent Text Processing.

[37] Meilicke C. Alignment incoherence in ontology matching. PhD thesis, Universität Mannheim. 2011.

[38] Scutellà MG (1990). A note on dowling and gallier’s top-down algorithm for propositional horn satisfiability. J Log Program.

[39] Chen X, Xia W, Jiménez-Ruiz E, Cross VV (2014). Extending an ontology alignment system with bioportal: a preliminary analysis. International Semantic Web Conference (Posters and Demos) 2014.

[40] Harrow I, Jiménez-Ruiz E, Splendiani A, Romacker M, Woollard P, Markel S, Alam-Faruque Y, Koch M, Malone J, Waaler A (2017). Matching disease and phenotype ontologies in the ontology alignment evaluation initiative. J Biomed Semant.

[41] PubMed. https://www.ncbi.nlm.nih.gov/pubmed. Accessed 04 Sept 2017.

[42] Cheatham M, Hitzler P (2013). String Similarity Metrics for Ontology Alignment.

[43] Cui L, Zhu W, Tao S, Case JT, Bodenreider O, Zhang GQ (2017). Mining non-lattice subgraphs for detecting missing hierarchical relations and concepts in snomed ct. J Am Med Inform Assoc.

[44] Zhu W, Zhang G-Q, Cui L (2017). Spark-mca: Large-scale, exhaustive formal concept analysis for evaluating the semantic completeness of snomed ct. AMIA Annual Symp Proc 2017.

